# Comparing Inpatient Satisfaction Collected via a Web-Based Questionnaire Self-Completion and Through a Telephone Interview: An Ancillary Study of the SENTIPAT Randomized Controlled Trial

**DOI:** 10.2196/jmir.7061

**Published:** 2017-08-23

**Authors:** Sarah F Feldman, Nathanael Lapidus, Jacques Cosnes, Emmanuel Tiret, Laurent Fonquernie, Jean Cabane, Olivier Chazouilleres, Laure Surgers, Marc Beaussier, Alain-Jacques Valleron, Fabrice Carrat, Gilles Hejblum

**Affiliations:** ^1^ Institut Pierre Louis d’Épidémiologie et de Santé Publique, Unité Mixte de Recherche en Santé 1136 Sorbonne Universités, Université Pierre et Marie Curie, Institut National de la Santé et de la Recherche Médicale Paris France; ^2^ Unité de Santé Publique Hôpital Saint-Antoine Assistance Publique–Hôpitaux de Paris Paris France; ^3^ Service de Gastro-Entérologie et Nutrition Hôpital Saint-Antoine Assistance Publique–Hôpitaux de Paris Paris France; ^4^ Service de Chirurgie Générale et Digestive Hôpital Saint-Antoine Assistance Publique–Hôpitaux de Paris Paris France; ^5^ Service de Maladies Infectieuses et Tropicales Hôpital Saint-Antoine Assistance Publique–Hôpitaux de Paris Paris France; ^6^ Service de Médecine Interne Hôpital Saint-Antoine Assistance Publique–Hôpitaux de Paris Paris France; ^7^ Service d'Hépato-Gastro-Entérologie Hôpital Saint-Antoine Assistance Publique–Hôpitaux de Paris Paris France; ^8^ Centre d'Immunologie et des Maladies Infectieuses, Unité Mixte de Recherche en Santé 1135 Sorbonne Universités, Université Pierre et Marie Curie, Institut National de la Santé et de la Recherche Médicale Paris France; ^9^ Service de Chirurgie Ambulatoire Hôpital Saint-Antoine Assistance Publique–Hôpitaux de Paris Paris France; ^10^ Thérapie Génique, Génétique, Epigénétique en Neurologie, Endocrinologie et Développement de l'Enfant, Unité Mixte de Recherche 1169 Université Paris-Sud, Institut National de la Santé et de la Recherche Médicale Le Kremlin-Bicêtre France

**Keywords:** hospital information systems, Internet, patient satisfaction, quality of health care, questionnaires and surveys, patient reported outcome measures, telephone

## Abstract

**Background:**

Assessing the satisfaction of patients about the health care they have received is relatively common nowadays. In France, the satisfaction questionnaire, I-Satis, is deployed in each institution admitting inpatients. Internet self-completion and telephone interview are the two modes of administration for collecting inpatient satisfaction that have never been compared in a multicenter randomized experiment involving a substantial number of patients.

**Objective:**

The objective of this study was to compare two modes of survey administration for collecting inpatient satisfaction: Internet self-completion and telephone interview.

**Methods:**

In the multicenter SENTIPAT (acronym for the concept of sentinel patients, ie, patients who would voluntarily report their health evolution on a dedicated website) randomized controlled trial, patients who were discharged from the hospital to home and had an Internet connection at home were enrolled between February 2013 and September 2014. They were randomized to either self-complete a set of questionnaires using a dedicated website or to provide answers to the same questionnaires administered during a telephone interview. As recommended by French authorities, the analysis of I-Satis satisfaction questionnaire involved all inpatients with a length of stay (LOS), including at least two nights. Participation rates, questionnaire consistency (measured using Cronbach alpha coefficient), and satisfaction scores were compared in the two groups.

**Results:**

A total of 1680 eligible patients were randomized to the Internet group (n=840) or the telephone group (n=840). The analysis of I-Satis concerned 392 and 389 patients fulfilling the minimum LOS required in the Internet and telephone group, respectively. There were 39.3% (154/392) and 88.4% (344/389) responders in the Internet and telephone group, respectively (P<.001), with similar baseline variables. Internal consistency of the global satisfaction score was higher (P=.03) in the Internet group (Cronbach alpha estimate=.89; 95% CI 0.86-0.91) than in the telephone group (Cronbach alpha estimate=.84; 95% CI 0.79-0.87). The mean global satisfaction score was lower (P=.03) in the Internet group (68.9; 95% CI 66.4-71.4) than in the telephone group (72.1; 95% CI 70.4-74.6), with a corresponding effect size of the difference at −0.253.

**Conclusions:**

The lower response rate issued from Internet administration should be balanced with a likely improved quality in satisfaction estimates, when compared with telephone administration, for which an interviewer effect cannot be excluded.

**Trial Registration:**

Clinicaltrials.gov NCT01769261 ; http://clinicaltrials.gov/ct2/show/NCT01769261 (Archived by WebCite at http://www.webcitation.org/6ZDF5lA41)

## Introduction

Numerous questionnaires have been developed since the 70s for assessing patient satisfaction with regard to hospital health care delivery. These include the Patient Satisfaction Questionnaire [1], the Client Satisfaction Questionnaire [2], the Service Quality instrument [3-5], the Hospital Consumer Assessment of Health Providers and Systems (HCAHPS) Survey [6,7], the Short Form HK Inpatient Experience Questionnaire [8,9], and the NHS National Adult Inpatient Survey [10]. In France, inpatient hospital satisfaction has been systematically measured since 2015 with the I-Satis questionnaire [11]. These numerous questionnaires attest a worldwide concern for enhancing the central role of the patient in the health care organization. Patient satisfaction assessment is also related to the technical performance and safety of hospital care [12] and is considered as a tool contributing to hospital care evaluation, although controversial [13]. Inpatient hospital satisfaction surveys are usually either self-administrated by pen and paper or conducted by telephone, with telephone interview being a common mode of questionnaire administration. However, the development of Internet has resulted in a widespread use of Web-based questionnaires, with corresponding survey costs lower than those of telephone surveys [14-17]. Moreover, the use of Internet has increased over time, with 78% of people with Internet access at home in France in 2013 [18], thereby suggesting that this mode of administration might result in a satisfactory response rate. Nevertheless, several studies have reported a lower response rate of Internet-based surveys, as compared with other modes of administration [19-21]. On the other hand, Internet self-completion has intrinsic favorable qualities such as the avoidance of any potential bias of responses related to an interviewer effect [22], and patients are more likely to freely express their opinions [23] on websites covering anonymity than through telephone. The way in which the modes of administration of patient satisfaction survey influence response rates and the issued scores remains an important issue. Several teams studied differences between pen-and-paper and Web-based questionnaires in the field of inpatient satisfaction and quality of life [21,24,25]; however, only a few investigated the differences with surveys administered through telephone [20,26], which remains a common mode of administration for inpatient hospital satisfaction surveys [27,28]. In this context, to our knowledge, this study—which is based on the multicenter SENTIPAT (sentinel patients) randomized trial [26,29]—is the first multicenter randomized trial to date comparing inpatient satisfaction collected via the Internet or through a telephone survey. Our objective was to assess whether response rates and satisfaction scores differed between these two modes of investigation of the patients' satisfaction.

## Methods

This research was an ancillary study of the multicenter, randomized SENTIPAT trial [29]. We took advantage of the trial to analyze patients’ satisfaction with their hospital stay.

### General Description of the SENTIPAT Trial

The SENTIPAT multicenter (five adult acute care units in a Parisian teaching hospital participated voluntarily: departments of digestive and general surgery; gastroenterology; hepatology; infectious diseases and tropical medicine; and internal medicine) randomized trial focused on the evolution of patients’ health on returning home post hospitalization (follow-up duration of 6 weeks). The general objective was to determine whether the information on patient’s health evolution shared by volunteer patients after returning home directly via a dedicated website was comparable with that obtained via telephone interviews. The randomization of 2050 patients (410 from each unit, 205 randomized in the Internet group and 205 randomized in the telephone group) was initially planned. The study was conducted in accordance with French regulation on ethics requirements in biomedical research.

Consecutive inpatients with Internet access at home were eligible for inclusion. Inclusion criteria also required inpatients who were not cognitively impaired and did not have a behavioral disorder, who spoke and wrote French, and were returning home after an acute care hospitalization, regardless of the type of stay—standard hospitalization (scheduled or not) on weekdays only (maximum Monday to Friday or any combination thereof) or outpatient hospitalization (1 day). Inpatients were enrolled on the day of hospital discharge by a clinical research technician of the trial. At that time, patients were informed about the study. Eligible patients not opposed to participate in the study were randomized into two parallel groups: Internet or telephone follow-up (inherently resulting in an open-label trial) at a ratio of 1:1. On the basis of a centralized randomization that allocated the eligible patient either to the Internet or to the telephone group through a website and using an underlying permutation block randomization stratified by service, the computer-generated list of permutation was established by a statistician independent from the study. At the time of patient inclusion, the technician also collected baseline variables (length of stay [LOS], sex, age, relationship status, level of education, activity, and type of insurance). Patient was then informed and discharged with documents explaining corresponding questionnaire administration.

### Characteristics of the Study

#### Patients

The French authorities have made available the instructions for analyzing I-Satis questionnaire [30], and according to these recommendations, the study was restricted to patients whose LOS included at least two consecutive nights.

#### Questionnaire Structure

The detailed I-Satis questionnaire used in this study (all questions and corresponding proposed answers) is directly accessible via the Internet [11]. The I-Satis questionnaire comprises 32 items exploring six dimensions: global care (Q1, Q2, Q4, Q13, Q14, and Q15), information to patient (Q16, Q18, Q27, Q28, Q29, and Q30), communication with health care providers (Q3, Q5, Q6, Q17, and Q20), behavior of health care providers (Q7, Q8, Q9, Q10, and Q11), hospital room convenience (Q21, Q22, Q23, and Q24), and hospital catering (Q25 and Q26). The recommendations of the French authorities for I-Satis analysis [30] indicate that 4 questions (Q12, Q19, Q31, and Q32) are not involved in score calculations.

#### Questionnaire Administration

All patients were informed that their opinions were kept anonymous. For the patients who had been randomized in the telephone group, the I-Satis questionnaire was administered during a telephone interview with a clinical research technician 7 days after discharge (the appointment was scheduled on the day of discharge), with a maximum of three attempts to contact them. For the patients who had been randomized in the Internet group, the same questionnaire was available on the dedicated website on the day of discharge (D0) and was completed directly online by the patient, who had been given oral and written instructions (information sheet) to connect for the first time 7 days post discharge. “Reminders” were sent by email once weekly for 6 weeks after discharge to potential responders of the Internet group, who had not completed the discharge questionnaire yet.

#### Score Construction

The questionnaires were analyzed according to the French national recommendations of the Direction Générale de l'Offre de Soins [30].

Each item was rated from 0 to 10. Rates 1 to 5 corresponded to increasing ordinal rankings of satisfaction; rates 0, 6, 7, and 8 corresponded to answers of nonrelevancy of the item for the patient (depending on the item: never felt discomfort, no drugs were prescribed, no surgery, etc), and spontaneous answers “I don’t know” and “I don’t wish to answer the question” were rated 9 and 10. Rates 1, 2, 3, 4, and 5 were valued 0, 25, 50, 75, and 100, respectively, in the analysis. Rates 0, 6, 7, 8, 9, and 10 were handled into the analyses as a missing value.

The scores of the dimensions “global care,” “information to patient,” “communication with health care providers,” “behavior of health care providers,” “hospital room convenience,” and “hospital catering” were calculated if at least three, three, three, three, two, and two items comprising the dimension were answered, respectively. The global score was calculated whenever every dimension score was calculated.

### Statistical Analysis

The participation rates observed in the Internet and telephone groups were compared using the Fisher exact test, as well as the proportions of nonrelevancy answers observed in these two groups. The delays of questionnaire completion observed in the Internet and telephone groups were compared using Wilcoxon-Mann-Whitney test. Internal consistency of questionnaires was measured by calculating Cronbach alpha [31], taking into account every score that could be calculated according to the abovementioned rules. An alpha coefficient value of greater than .7 was considered as satisfactory. Dimensions’ scores were calculated for each patient as the mean of the corresponding dimensions’ items, and global score was the mean of all answered items of the questionnaire. CIs were obtained by bootstrap. Standardized Cohen *d*-type effect size was measured between the scores of the two groups [32].

Comparisons between the Internet and telephone groups in terms of Cronbach alpha coefficients and in terms of satisfaction scores (including dimensions’ scores) were made using a permutation test [33], with the null hypothesis distribution (distribution of the difference between the two groups under the hypothesis of no difference) generated through 1,000,000 shuffled datasets. A *P* value of ≤.05 defined the significance of comparisons.

Missing data were handled as follows: First, nonresponding patients were excluded from score analyses. Patients for whom less than 16 items were completed were also excluded from score analyses (ie, handled as nonresponders in the analyses). Second, the scores issued from the remaining partially completed questionnaires were calculated as mentioned above (see subsection on Score Construction).

All analyses were made with the R statistical computing freeware version 3.3.0 [34].

### Ethic and Legal Approvals

The SENTIPAT study was approved by the Comité de Protection des Personnes Ile de France IX (decision CPP-IDF IX 12-014, June 12, 2012); the Comité Consultatif sur le Traitement de l'Information en matière de Recherche dans le domaine de la Santé (Decision 12.365, June 20, 2012); and the Commission Nationale de l’Informatique et des Libertés (Decision DR-2012-582, December 12, 2012).

## Results

**Figure 1:**
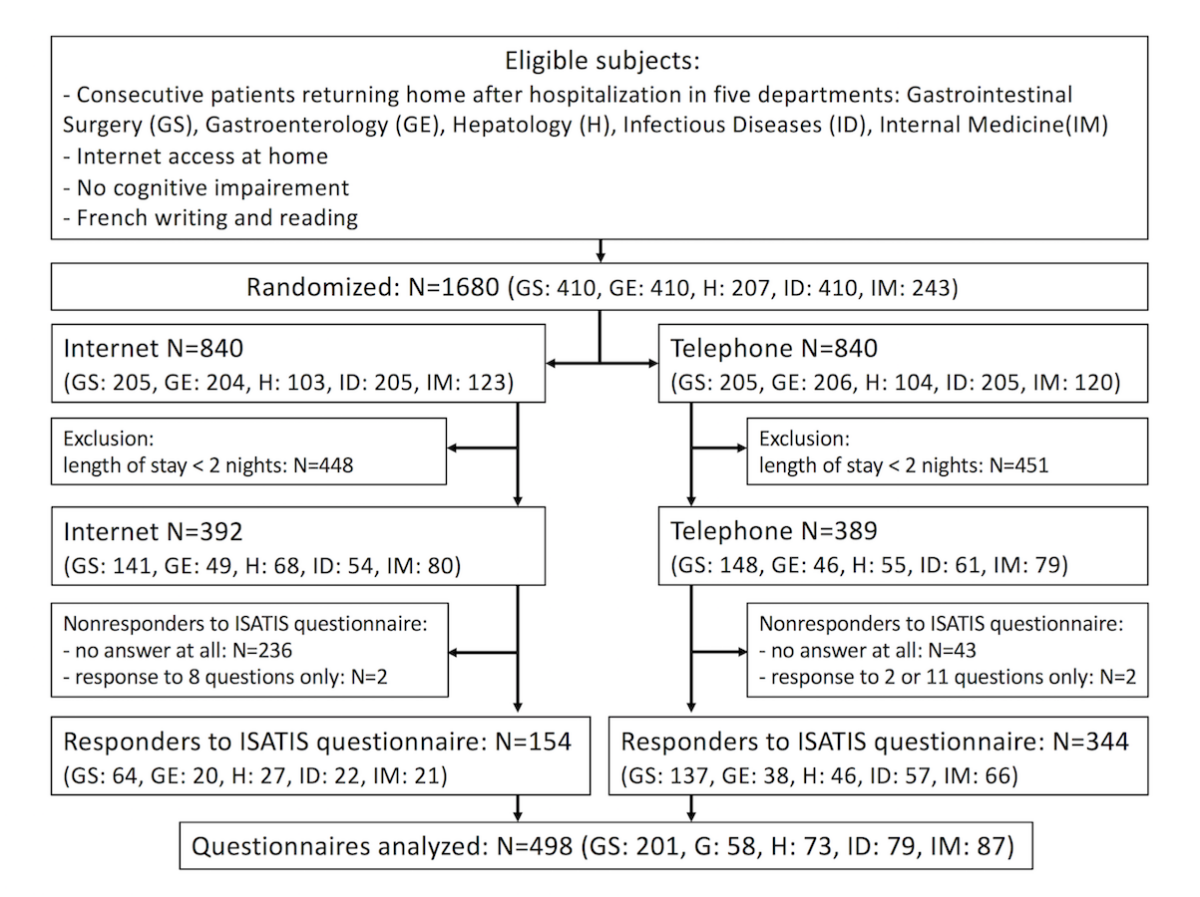
Flow of participants through the study.

Between February 25, 2013 and September 8, 2014, we managed to enroll in the SENTIPAT study 1680 eligible patients (840 randomized in the Internet group and telephone group each) and not opposed to participating in the trial. Among these, the baseline population of patients fulfilling the minimum LOS of 2 nights required for I-Satis investigation comprised 781 patients, with 392 and 389 patients in the Internet and telephone groups, respectively (Figure 1). Table 1 provides the details of baseline values of the patients who constituted the population investigated in this study. There were no missing data relating to baseline values. Considering all 781 patients, the median LOS was 5 days (interquartile range [IQR]: 2-9); there were as many men as women, participants were aged 19 to 97 years, and median age was 53 years (IQR: 37-64), and 711 patients (91.0%) had a complementary private health insurance in addition to the compulsory health insurance.

There were 154 responders out of the 392 patients in the Internet group (response rate of 39.3%) and 344 responders out of the 389 patients in the telephone group (response rate of 88.4%; *P*<.001), and the corresponding median delays between hospital discharge and questionnaire completion were 6 days (IQR: 3-15.75) and 7 days (IQR: 7-9), respectively (*P*=.002). Missing data in responders concerned 10 patients of the Internet group: answer to question 13 (satisfaction about pain management) was missing in 2 responders, answer to question 20 (satisfaction about the answers of the surgeon about patient’s questions on surgery) was missing in 3 responders, and answer to both questions was missing in 5 responders. In addition, there were 13 (8.4%), 95 (61.7%), 43 (27.9%), and 3 (1.9%) responders in the Internet group with 0, 1 to 5, 6 to 10, and more than 10 answers, for which the answer code corresponded to nonrelevancy or refusal (further handled as a missing value in the analyses, see the section on Methods), respectively, whereas the corresponding responders observed in the telephone group were 3 (0.8%), 124 (36.0%), 200 (58.1%), and 15 (4.4%), respectively. Internet responders provided an answer code corresponding to nonrelevancy and refusal less frequently than telephone responders (*P*<.001).

**Table 1:** Characteristics of study patients.

Variable		Total, N=781	Responders Internet, n=154	Nonresponders Internet, n=238	Responders telephone, n=344	Nonresponders telephone, n=45
LOS^a^, in days, median (IQR^b^)		5 (2-9)	5 (3-8)	5.5 (3-8)	5 (2-9)	7 (3-10)
**Sex, n (%)**						
	Male	385 (49.4)	71 (46.1)	115 (48.3)	176 (51.2)	23 (51)
Age in years, median (IQR)		53 (37-64)	55 (38-64)	50 (36-64)	53 (38-65)	51 (33-67)
**Relationship status, n (%)**						
	Living alone^c^	342 (43.8)	59 (38.3)	112 (47.1)	146 (42.4)	25 (56)
	Living as a couple^d^	439 (56.2)	95 (61.7)	126 (52.9)	198 (57.6)	20 (44)
**Level of education, n (%)**						
	Lower secondary education	88 (11.3)	17 (11.0)	28 (11.8)	35 (10.2)	8 (18)
	Upper secondary education	249 (32.0)	46 (29.9)	80 (33.8)	110 (32.0)	13 (30)
	Postsecondary nontertiary education or short-cycle tertiary	113 (14.5)	23 (14.9)	32 (13.5)	53 (15.4)	5 (11)
	Bachelor’s degree or above	329 (42.2)	68 (44.2)	97 (40.9)	146 (42.4)	18 (41)
**Activity, n (%)**						
	Nonworking	321 (41.2)	64 (42.1)	101 (42.4)	139 (40.4)	17 (38)
	Employed or student	458 (58.8)	88 (57.9)	137 (57.6)	205 (59.6)	28 (62)
**Insurance, n (%)**						
	Precarious^e^	30 (03.8)	3 (02.0)	13 (05.5)	11 (03.2)	3 (07)
	Compulsory health insurance	40 (05.1)	7 (04.6)	15 (06.3)	14 (04.1)	4 (09)
	Compulsory health insurance plus complementary private health insurance	711 (91.0)	144 (93.5)	210 (88.2)	319 (92.7)	38 (84)
Delay between hospital discharge and questionnaire completion, in days, median (IQR)		N/A^f^	6 (3-15.75)	N/A	7 (7-9)	N/A

^a^LOS: length of hospital stay.

^b^IQR: interquartile range.

^c^Single, widowed, divorced, separated.

^d^Married, living together under a civil solidarity pact, simply living together without legal ties.

^e^Benefit from state medical help or universal health insurance.

^f^N/A: not applicable.

Table 2 shows the values of Cronbach alpha in the Internet and telephone responders. All estimates, with the exception of those corresponding to room convenience, were >.7. The alpha estimates observed in the Internet group were always greater than those observed in the telephone group, the difference being statistically significant for the two dimensions, global care and room convenience (*P*=.003 and *P*=.03, respectively), and for the global satisfaction (*P*=.03).

Table 3 summarizes the satisfaction scores observed in the Internet and telephone groups. The mean global satisfaction score was 68.89 (95% CI 66.36-71.36) in the Internet group and 72.01 (95% CI 70.36-73.58) in the telephone group. In both groups, the dimension that received the lowest score was hospital catering, with means of 45.77 (95% CI 42.18-49.39) and 45.70 (95% CI 43.32-48.06) in the Internet and telephone group, respectively. Conversely, in both groups, the theme that received the highest score was behavior of health care providers, with means of 87.49 (95% CI 85.05-89.73) and 92.14 (95% CI 90.81-93.39) in the Internet and telephone group, respectively. There were three dimension scores significantly smaller in the Internet group than in the telephone group: information to patients with a mean difference of −5.38 (*P*=.008), communication with health care providers with a mean difference of −7.16 (*P*=.003), and behavior of health care providers with a mean of −4.66 (*P*<.001). The global satisfaction score was significantly smaller in the Internet group with a mean difference of −7.16 (*P*=.003) and behavior of health care providers with a mean of −4.66 (*P*<.001). The global satisfaction score was significantly smaller in the Internet group, with a mean difference of −3.12 (*P*=.03). The absolute values of the effect sizes ranged from 0.003 to 0.352. The satisfaction scores observed in the group of Internet responders according to the delay of questionnaire completion are summarized in Multimedia Appendix 1, and whatever the type of score considered, the score difference between the two subgroups of Internet responders (questionnaire completed at day 7 after discharge or later versus questionnaire completed earlier) was not significant.

**Table 2:** Cronbach alpha coefficients.

Dimension of the score (number of items involved)	Internet responders: Cronbach alpha (95% CI); n	Telephone responders: Cronbach alpha (95% CI); n	Comparison between the two groups: *P* value
Global care (6)	.92 (0.87-0.96); 154	.79 (0.73-0.85); 344	.003
Information to patients (6)	.89 (0.81-0.94); 131	.83 (0.76-0.88); 235	.27
Communication with health care providers (5)	.74 (0.65-0.82); 152	.71 (0.64-0.77); 334	.56
Behavior of health care providers (5)	.82 (0.71-0.92); 153	.73 (0.61-0.84); 344	.33
Hospital room convenience (4)	.66 (0.53-0.75); 154	.48 (0.37-0.56); 344	.03
Hospital catering (2)	.86 (0.79-0.91); 136	.76 (0.69-0.82); 285	.07
Global satisfaction score (28)	.89 (0.86-0.91); 116	.84 (0.79-0.87); 189	.03

**Table 3:** Satisfaction scores.

Dimension of the score	Internet responders: mean score (95% CI); n	Telephone responders: mean score (95% CI); n	Internet-telephone: mean score difference (95% CI), *P* value	Effect size (95% CI)
Global care	70.67 (68.01-73.34); 154	72.15 (70.56-73.75); 344	−1.48 (−4.59 to 1.64), .33	−0.094 (−0.290 to 0.105)
Information to patients	59.62 (56.07-63.23); 131	65.01 (62.75-67.25); 235	−5.38 (−9.53 to −1.19), .009	−0.286 (−0.501 to −0.062)
Communication with health care providers	67.42 (63.28-71.42); 152	74.58 (71.90-77.15); 334	−7.16 (−12.01 to −2.36), .003	−0.287 (−0.477 to −0.096)
Behavior of health care providers	87.49 (85.05-89.73); 153	92.14 (90.81-93.39); 344	−4.66 (−7.40 to −2.02), <.001	−0.352 (−0.554 to −0.154)
Hospital room convenience	61.03 (58.07-63.95); 154	60.97 (59.07-62.88); 344	0.05 (−3.46 to 3.50), .98	0.003 (−0.189 to 0.195)
Hospital catering	45.77 (42.18-49.39); 136	45.70 (43.32-48.06); 285	0.07 (−4.24 to 4.44), .98	0.003 (−0.206 to 0.214]
Global satisfaction score	68.89 (66.36-71.36); 116	72.01 (70.36-73.58); 189	−3.12 (−6.13 to −0.15), .03	−0.253 (−0.490 to −0.014]

## Discussion

### Principal Findings

The investigation of patient satisfaction after a hospital stay resulted in several differences when comparing the two modes of questionnaire administration: self-reported Internet completion or telephone interview. The comparison between these modes of administration may be discussed according to three topics: response rate, questionnaire reliability, and satisfaction scores. The response rate observed in the group of patients randomized in the Internet group (39.3%) was much lower than that observed in the group of patients randomized in the telephone group (88.4%). Such a difference might have resulted in unbalancing the initial comparability of responders in the two groups even if Table 1 indicates that baseline variables are similar in the responders of the two groups. Unsurprisingly, the observed difference between the two groups in terms of participation rate is in accordance with the previous results issued from the same cohort focusing on patient satisfaction with regard to the hospital discharge process [26]. The difference between the participation rates observed with the two administration modes of the survey might be, at least in part, owing to the fact that it is easier to ignore an email than a phone call scheduled at a date chosen by the patient. The participation rates observed in our study are also similar to those reported by Harewood et al [20] who investigated patient satisfaction with endoscopy and observed a response rate of 34% and 78% in the Internet group and telephone group, respectively. However, comparing the response rates observed in our study with other rates previously reported is probably of limited interest as the study design widely varies from one study to another, as response rates are likely to be highly sensitive to the detailed underlying procedures for selecting participants (eg, face-to-face enrollment vs random selection in an administrative database, issues related to the initial comparability of the participants allocated in the Internet and telephone arms), reaching/soliciting responders (including reminding procedures for soliciting Web participants to complete the survey or procedure for scheduling the phone calls), and collecting answers (eg, attractiveness of the website and ease in accessing/completing the questionnaire form).

With the exception of the values for the hospital room convenience dimension, which raise concerns, the values of Cronbach alpha were satisfactory for all dimensions investigated and for the global satisfaction score, favoring the conduction of surveys with this questionnaire using either administration mode. Besides, interestingly, considering all six dimensions of the questionnaire, the values of Cronbach alpha were always higher in the Internet group than in the telephone group, with a statistical significance observed for two dimensions (global care and hospital room convenience) and for the global satisfaction score. Here, the adjunction of an interviewer in the telephone group (as compared with self-completion in the Internet group) might be considered as an undesired burden disturbing initial signal.

The observed score differences between the Internet and telephone groups (see Table 3) are contrasted, depending on the dimension investigated. On the one hand, considering hospital room convenience and hospital catering dimensions, both telephone and Internet modes of administration resulted in very similar satisfaction scores, and the difference was only slight when considering global care dimension. On the other hand, in the three dimensions related to interactions with health care providers, that is, information to patients, communication with health care providers, and behavior of health care providers, scores were significantly lower in the Internet group than in the telephone group, although it is worth mentioning that corresponding effect sizes never exceeded 0.35, a value below the medium threshold proposed by Cohen [32]. Such an observation raises a general comment on the surveys conducted in this domain. Those surveys are deployed for investigating patient satisfaction with hospital services, for bringing into light the elements which require improvements and for assessing evolution with time. Additionally, French authorities require the analysis of 120 patients per medical center each year. In such a context, our study’s finding that a mean difference of 7 points based on a sample size of 498 responders is modest in terms of effect size, suggests that potential improvements on patient satisfaction are very difficult to evidence. Dynamic trends within a given center from one year to another should be interpreted with great caution and must take into account the underlying variability of the scores, and a similar caution should be required in the interpretation of differences between centers. A potential explanation for the higher scores observed in the telephone group is that a patient might be more reluctant to provide low scores to an interviewer (moreover potentially identified as a member of the hospital staff) than when completing a strict anonymous form via the Internet. Previous studies [35,36] have also mentioned such a social desirability bias [23] as a potential explanation for the higher patient satisfaction scores issued from a questionnaire administered by a telephone interviewer as compared with a self-completed form administered by mail [35] or via the Internet [36]. In addition, the distribution of the delay between hospital discharge and questionnaire completion was more variable in the Internet group. However, as shown in the Multimedia Appendix 1, the scores of the Internet responders did not significantly vary according to the delay of questionnaire completion, indicating that the wider variability in the delay of questionnaire completion observed in the Internet group had a very limited impact (if any) on the differences of scores that were observed between the telephone and Internet modes of administration.

### Strengths and Limitations

A strength of the study relies on the fact that it is the first randomized trial reported to date that compared inpatient satisfaction collected either via a telephone interview or via the self-completion of a similar questionnaire on a dedicated website and involved a reasonable sample of inpatients, both in terms of case-mix variability (patients originating from 5 very different hospital wards) and in terms of sample size (498 questionnaires were eventually analyzed). A home access to the Internet and a phone number were two required inclusion criteria for patient eligibility, ensuring the initial comparability of the individuals randomized in the two administration modes of the questionnaire. Moreover, to our knowledge, this study is the first to date that explores, in detail, critical issues relating to the I-Satis questionnaire, which is dedicated to be deployed in all inpatient structures in France; on the one hand, this constitutes an additional strength of the study, while on the other hand, the fact that this questionnaire is yet restricted to France constitutes a limitation of the study.

### Conclusions

In conclusion, our study shows that the lower response rate observed with the Internet mode of administration than that observed with the telephone mode of administration must be balanced with other positive features associated with the Internet. Using the latter mode of administration has a potential lower cost than telephone [14-17], and the quality of satisfaction estimates is likely improved because the potential veil of a telephone interviewer is discarded, allowing patients to express more freely. They are more likely to rate their satisfaction about hospital stay with lower scores. This study indicates that some of these score decreases are statistically significant but the corresponding effect sizes are small, indicating that the decreases relate to moderate differences. Communication habits are evolving, and the Web form is progressively adopted as a reference mode for administrating surveys as well as a reference mode for completing questionnaires. With the exception of the higher response rate observed with telephone interview in this study, all other study results support the deployment of Web-based questionnaires for exploring inpatient satisfaction.

## Appendix

Multimedia Appendix 1 Satisfaction scores observed in the Internet responder group according to the delay of questionnaire completion.

Multimedia Appendix 2 CONSORT-EHEALTH checklist (v.1.6.1).

## Abbreviations

HCAHPS: Hospital Consumer Assessment of Health Providers and Systems
IQR: interquartile range
LOS: length of stay
