# Supplementary material for: Comparing Inpatient Satisfaction Collected via a Web-Based Questionnaire Self-Completion and Through a Telephone Interview: An Ancillary Study of the SENTIPAT Randomized Controlled Trial
Source: J Med Internet Res. 2017 Aug 23;19(8):e293. doi: 10.2196/jmir.7061 (PMC5587887; doi:10.2196/jmir.7061)
Supplement: Multimedia Appendix 1 [file jmir_v19i8e293_app1.pdf]

MULTIMEDIA APPENDIX 1

Table A1-1. Satisfaction scores observed in the Internet responder group according to the delay of questionnaire completion: responders who completed the questionnaire at day seven after hospital discharge or later (late responders), and responders who completed the questionnaire within a delay of six days after hospital discharge (early responders).

Dimension of the score	Internet late responders: Mean score [95%CI]; n	Internet early responders: Mean score [95%CI]; n	Late - early: Mean score difference [95%CI], P value	Effect size [95%CI]
Global care	72.37 [68.51-76.22]; 73	69.14 [65.50-72.73]; 81	3.23 [−2.14 to 8.54], .24	−0.192 [−0.505 to 0.129]
Information to patients	59.92 [54.99-64.81]; 65	59.34 [54.08-64.55]; 66	0.58 [−6.55 to 7.69], .87	−0.028 [−0.370 to 0.318]
Communication with healthcare providers	69.62 [63.57-75.28]; 72	65.44 [59.82-71.08]; 80	4.18 [−4.01 to 12.21], .32	−0.164 [−0.480 to 0.158]
Behavior of healthcare providers	88.11 [85.09-90.94]; 73	86.92 [83.15-90.39]; 80	1.19 [−3.35 to 5.97], .62	−0.082 [−0.373 to 0.246]
Hospital room convenience	61.62 [57.28-65.78]; 73	60.49 [56.57-64.52]; 81	1.13 [−4.68 to 6.97], .71	−0.061 [−0.385 to 0.257]
Hospital catering	43.95 [38.44-49.54]; 64	47.40 [42.72-52.17]; 72	−3.45 [−10.63 to 3.84], .37	0.161 [−0.173 to 0.493]
Global satisfaction score	69.22 [65.80-72.67]; 57	68.56 [64.90-72.20]; 59	0.66 [−4.35 to 5.67], .80	−0.047 [−0.416 to 0.319]
