# Supplementary material for: Comparing Inpatient Satisfaction Collected via a Web-Based Questionnaire Self-Completion and Through a Telephone Interview: An Ancillary Study of the SENTIPAT Randomized Controlled Trial
Source: J Med Internet Res. 2017 Aug 23;19(8):e293. doi: 10.2196/jmir.7061 (PMC5587887; doi:10.2196/jmir.7061)
Supplement: Multimedia Appendix 2 [file jmir_v19i8e293_app2.pdf]

CONSORT-EHEALTH (V 1.6.1) - Submission/Publication Form

The CONSORT-EHEALTH checklist is intended for authors of randomized trials evaluating web-based and Internet-based applications/interventions, including mobile interventions, electronic games (incl multiplayer games), social media, certain telehealth applications, and other interactive and/or networked electronic applications. Some of the items (e.g. all subitems under item 5 - description of the intervention) may also be applicable for other study designs.

The goal of the CONSORT EHEALTH checklist and guideline is to be
a) a guide for reporting for authors of RCTs,
b) to form a basis for appraisal of an ehealth trial (in terms of validity)

CONSORT-EHEALTH items/subitems are MANDATORY reporting items for studies published in the Journal of Medical Internet Research and other journals / scientific societies endorsing the checklist.

Items numbered 1., 2., 3., 4a., 4b etc are original CONSORT or CONSORT-NPT (non-pharmacologic treatment) items.
Items with Roman numerals (i., ii, iii, iv etc.) are CONSORT-EHEALTH extensions/clarifications.

As the CONSORT-EHEALTH checklist is still considered in a formative stage, we would ask that you also RATE ON A SCALE OF 1-5 how important/useful you feel each item is FOR THE PURPOSE OF THE CHECKLIST and reporting guideline (optional).

Mandatory reporting items are marked with a red *.
In the textboxes, either copy & paste the relevant sections from your manuscript into this form - please include any quotes from your manuscript in QUOTATION MARKS,
or answer directly by providing additional information not in the manuscript, or elaborating on why the item was not relevant for this study.

YOUR ANSWERS WILL BE PUBLISHED AS A SUPPLEMENTARY FILE TO YOUR PUBLICATION IN JMIR AND ARE CONSIDERED PART OF YOUR PUBLICATION (IF ACCEPTED).
Please fill in these questions diligently. Information will not be copyedited, so please use proper spelling and grammar, use correct capitalization, and avoid abbreviations.

DO NOT FORGET TO SAVE AS PDF _AND_ CLICK THE SUBMIT BUTTON SO YOUR ANSWERS ARE IN OUR DATABASE !!!

Citation Suggestion (if you append the pdf as Appendix we suggest to cite this paper in the caption):
Eysenbach G, CONSORT-EHEALTH Group
CONSORT-EHEALTH: Improving and Standardizing Evaluation Reports of Web-based and Mobile Health Interventions
J Med Internet Res 2011;13(4):e126
URL: http://www.jmir.org/2011/4/e126/
doi: 10.2196/jmir.1923
PMID: 22209829

*Obligatoire

Your name *

First Last

Gilles Hejblum

Primary Affiliation (short), City, Country *

University of Toronto, Toronto, Canada

Inserm, Paris, France

Your e-mail address *

abc@gmail.com

gilles.hejblum@inserm.fr

Title of your manuscript *

Provide the (draft) title of your manuscript.

Comparing inpatient satisfaction collected via a web-based questionnaire self-completion and through a telephone interview: an ancillary study of the SENTIPAT randomized controlled trial

Article Preparation Status/Stage *

At which stage in your article preparation are you currently (at the time you fill in this form)

- ○ not submitted yet - in early draft status
- ● not submitted yet - in late draft status, just before submission
- ○ submitted to a journal but not reviewed yet
- ○ submitted to a journal and after receiving initial reviewer comments
- ○ submitted to a journal and accepted, but not published yet
- ○ published
- ○ Autre :

Journal *

If you already know where you will submit this paper (or if it is already submitted), please provide the journal name (if it is not JMIR, provide the journal name under "other")

- ○ not submitted yet / unclear where I will submit this
- ● Journal of Medical Internet Research (JMIR)
- ○ Autre :

Manuscript tracking number *

If this is a JMIR submission, please provide the manuscript tracking number under "other" (The ms tracking number can be found in the submission acknowledgement email, or when you login as author in JMIR. If the paper is already published in JMIR, then the ms tracking number is the four-digit number at the end of the DOI, to be found at the bottom of each published article in JMIR)

- ● no ms number (yet) / not (yet) submitted to / published in JMIR
- ○ Autre :

TITLE AND ABSTRACT

1a) TITLE: Identification as a randomized trial in the title

1a) Does your paper address CONSORT item 1a? *

I.e does the title contain the phrase "Randomized Controlled Trial"? (if not, explain the reason under "other")

- (x) yes
- ( ) Autre :

1a-i) Identify the mode of delivery in the title

Identify the mode of delivery. Preferably use "web-based" and/or "mobile" and/or "electronic game" in the title. Avoid ambiguous terms like "online", "virtual", "interactive". Use "Internet-based" only if Intervention includes non-web-based Internet components (e.g. email), use "computer-based" or "electronic" only if offline products are used. Use "virtual" only in the context of "virtual reality" (3-D worlds). Use "online" only in the context of "online support groups". Complement or substitute product names with broader terms for the class of products (such as "mobile" or "smart phone" instead of "iphone"), especially if the application runs on different platforms.

	1	2	3	4	5	
subitem not at all important	( )	( )	( )	( )	(x)	essential

Does your paper address subitem 1a-i? *

Copy and paste relevant sections from manuscript title (include quotes in quotation marks "like this" to indicate direct quotes from your manuscript), or elaborate on this item by providing additional information not in the ms, or briefly explain why the item is not applicable/relevant for your study

"web-based questionnaire"

1a-ii) Non-web-based components or important co-interventions in title

Mention non-web-based components or important co-interventions in title, if any (e.g., "with telephone support").

	1	2	3	4	5	
subitem not at all important	( )	( )	( )	( )	(x)	essential

Does your paper address subitem 1a-ii?

Copy and paste relevant sections from manuscript title (include quotes in quotation marks "like this" to indicate direct quotes from your manuscript), or elaborate on this item by providing additional information not in the ms, or briefly explain why the item is not applicable/relevant for your study

"telephone interview"

1a-iii) Primary condition or target group in the title

Mention primary condition or target group in the title, if any (e.g., “for children with Type I Diabetes”) Example: A Web-based and Mobile Intervention with Telephone Support for Children with Type I Diabetes: Randomized Controlled Trial

	1	2	3	4	5	
subitem not at all important	○	○	○	○	●	essential

Does your paper address subitem 1a-iii? *

Copy and paste relevant sections from manuscript title (include quotes in quotation marks "like this" to indicate direct quotes from your manuscript), or elaborate on this item by providing additional information not in the ms, or briefly explain why the item is not applicable/relevant for your study

"inpatient satisfaction"

1b) ABSTRACT: Structured summary of trial design, methods, results, and conclusions

NPT extension: Description of experimental treatment, comparator, care providers, centers, and blinding status.

1b-i) Key features/functionalities/components of the intervention and comparator in the METHODS section of the ABSTRACT

Mention key features/functionalities/components of the intervention and comparator in the abstract. If possible, also mention theories and principles used for designing the site. Keep in mind the needs of systematic reviewers and indexers by including important synonyms. (Note: Only report in the abstract what the main paper is reporting. If this information is missing from the main body of text, consider adding it)

	1	2	3	4	5	
subitem not at all important	○	○	○	○	●	essential

Does your paper address subitem 1b-i? *

Copy and paste relevant sections from the manuscript abstract (include quotes in quotation marks "like this" to indicate direct quotes from your manuscript), or elaborate on this item by providing additional information not in the ms, or briefly explain why the item is not applicable/relevant for your study

"In the multicentre SENTIPAT randomized trial, patients discharged from the hospital to home and having an internet connection at home were enrolled between February 2013 and September 2014. They were randomized to either selfcomplete a set of questionnaires using a dedicated website (I group, n=840) or to provide answers during a telephone interview (T group, n=840)."

1b-ii) Level of human involvement in the METHODS section of the ABSTRACT

Clarify the level of human involvement in the abstract, e.g., use phrases like “fully automated” vs. “therapist/nurse/care provider/physician-assisted” (mention number and expertise of providers involved, if any). (Note: Only report in the abstract what the main paper is reporting. If this information is missing from the main body of text, consider adding it)

	1	2	3	4	5	
subitem not at all important	○	○	○	○	○	essential

Does your paper address subitem 1b-ii?

Copy and paste relevant sections from the manuscript abstract (include quotes in quotation marks "like this" to indicate direct quotes from your manuscript), or elaborate on this item by providing additional information not in the ms, or briefly explain why the item is not applicable/relevant for your study

"They were randomized to either self-complete a set of questionnaires using a dedicated website or to provide answers to the same questionnaires administered during a telephone interview."

1b-iii) Open vs. closed, web-based (self-assessment) vs. face-to-face assessments in the METHODS section of the ABSTRACT

Mention how participants were recruited (online vs. offline), e.g., from an open access website or from a clinic or a closed online user group (closed usergroup trial), and clarify if this was a purely web-based trial, or there were face-to-face components (as part of the intervention or for assessment). Clearly say if outcomes were self-assessed through questionnaires (as common in web-based trials). Note: In traditional offline trials, an open trial (open-label trial) is a type of clinical trial in which both the researchers and participants know which treatment is being administered. To avoid confusion, use “blinded” or “unblinded” to indicated the level of blinding instead of “open”, as “open” in web-based trials usually refers to “open access” (i.e. participants can self-enrol). (Note: Only report in the abstract what the main paper is reporting. If this information is missing from the main body of text, consider adding it)

	1	2	3	4	5	
subitem not at all important	○	○	○	○	○	essential

Does your paper address subitem 1b-iii?

Copy and paste relevant sections from the manuscript abstract (include quotes in quotation marks "like this" to indicate direct quotes from your manuscript), or elaborate on this item by providing additional information not in the ms, or briefly explain why the item is not applicable/relevant for your study

"patients discharged from the hospital to home and having an internet connection at home were enrolled" ...
"They were randomized to either self-complete a set of questionnaires using a dedicated website or to provide answers to the same questionnaires administered during a telephone interview."

1b-iv) RESULTS section in abstract must contain use data

Report number of participants enrolled/assessed in each group, the use/uptake of the intervention (e.g., attrition/adherence metrics, use over time, number of logins etc.), in addition to primary/secondary outcomes. (Note: Only report in the abstract what the main paper is reporting. If this information is missing from the main body of text, consider adding it)

	1	2	3	4	5	
subitem not at all important	○	○	○	○	○	essential

Does your paper address subitem 1b-iv?

Copy and paste relevant sections from the manuscript abstract (include quotes in quotation marks "like this" to indicate direct quotes from your manuscript), or elaborate on this item by providing additional information not in the ms, or briefly explain why the item is not applicable/relevant for your study

"1680 eligible patients were randomized in the Internet group (n=840) or Telephone group (n=840). Analysis of I-Satis concerned 392 and 389 patients in the Internet and Telephone group fulfilling the minimum length of stay required. There were 154 (39%) and 344 (88%) responders in the Internet and Telephone group, respectively (P<.001), with similar baseline variables. Internal consistency of the global satisfaction score was higher (P=.034) in the Internet group (Cronbach alpha estimate [95% confidence interval]: 0.89 [0.86–0.91]) than in the Telephone group (0.84 [0.79–0.87]). The mean global satisfaction score [95% confidence interval] was lower (P=.032) in the Internet group (68.9 [66.4–71.4]) than in the Telephone group (72.1 [70.4–74.6]), with a

1b-v) CONCLUSIONS/DISCUSSION in abstract for negative trials

Conclusions/Discussions in abstract for negative trials: Discuss the primary outcome - if the trial is negative (primary outcome not changed), and the intervention was not used, discuss whether negative results are attributable to lack of uptake and discuss reasons. (Note: Only report in the abstract what the main paper is reporting. If this information is missing from the main body of text, consider adding it)

	1	2	3	4	5	
subitem not at all important	○	○	○	○	○	essential

Does your paper address subitem 1b-v?

Copy and paste relevant sections from the manuscript abstract (include quotes in quotation marks "like this" to indicate direct quotes from your manuscript), or elaborate on this item by providing additional information not in the ms, or briefly explain why the item is not applicable/relevant for your study

Not Applicable: positive and negative meanings do not apply to this ancillary study.
"The lower response rate issued from Internet administration should be balanced with a likely improved quality in satisfaction estimates, when compared to Telephone administration for which an interviewer effect cannot be excluded."

INTRODUCTION

2a) In INTRODUCTION: Scientific background and explanation of rationale

2a-i) Problem and the type of system/solution

Describe the problem and the type of system/solution that is object of the study: intended as stand-alone intervention vs. incorporated in broader health care program? Intended for a particular patient population? Goals of the intervention, e.g., being more cost-effective to other interventions, replace or complement other solutions? (Note: Details about the intervention are provided in

“Methods” under 5)

	1	2	3	4	5	
subitem not at all important	○	○	○	○	○	essential

Does your paper address subitem 2a-i? *

Copy and paste relevant sections from the manuscript (include quotes in quotation marks "like this" to indicate direct quotes from your manuscript), or elaborate on this item by providing additional information not in the ms, or briefly explain why the item is not applicable/relevant for your study

"How modes of administration of patient satisfaction survey influence response rates and the issued scores remains an important issue."
...
"only few investigated the differences with surveys administered through telephone, while this mode of administration remains common for inpatient hospital satisfaction surveys [...] to our knowledge, the present study [...] is the first multicentre randomized

2a-ii) Scientific background, rationale: What is known about the (type of) system

Scientific background, rationale: What is known about the (type of) system that is the object of the study (be sure to discuss the use of similar systems for other conditions/diagnoses, if appropiate), motivation for the study, i.e. what are the reasons for and what is the context for this specific study, from which stakeholder viewpoint is the study performed, potential impact of findings [2]. Briefly justify the choice of the comparator.

	1	2	3	4	5	
subitem not at all important	○	○	○	○	○	essential

Does your paper address subitem 2a-ii? *

Copy and paste relevant sections from the manuscript (include quotes in quotation marks "like this" to indicate direct quotes from your manuscript), or elaborate on this item by providing additional information not in the ms, or briefly explain why the item is not applicable/relevant for your study

"Inpatient hospital satisfaction surveys are usually either self-administrated by pen-and-paper or conducted by telephone, the telephone interview being a common mode of questionnaire administration. However, the development of internet has resulted in a large spreading of online questionnaires, with corresponding survey costs lower than those of mail questionnaires, and also less time consuming [14,15]. Moreover, the use of internet has increased with time, with for example, 78% of people that had an internet access at home in France in 2013 [16], suggesting that this mode of administration might result in a satisfactory response rate. Nevertheless, several studies have reported a lower response rate of internet-based surveys, as compared to other modes of administration.[17-19]. On the other hand, internet self-completion has intrinsic favorable qualities such as the avoidance of any potential bias of responses related to an interviewer effect [20], and patients are likely more free to express their opinions [21] on web-sites covering anonymity than through telephone. How modes of administration of patient satisfaction survey influence response rates and the issued scores remains an important issue. Several teams studied differences between pen-and-paper and online questionnaires in the field of inpatient satisfaction and quality of life [15,19,22], but only few investigated the differences with surveys administered through telephone [18,23], while this mode of administration remains common for inpatient hospital satisfaction surveys [24,25]. In this context, to our knowledge, the present study

2b) In INTRODUCTION: Specific objectives or hypotheses

Does your paper address CONSORT subitem 2b? *

Copy and paste relevant sections from the manuscript (include quotes in quotation marks "like this" to indicate direct quotes from your manuscript), or elaborate on this item by providing additional information not in the ms, or briefly explain why the item is not applicable/relevant for your study

"Our objective was to assess whether response rates and satisfaction scores differed between these two modes of investigation of the patients' satisfaction."

METHODS

3a) Description of trial design (such as parallel, factorial) including allocation ratio

Does your paper address CONSORT subitem 3a? *

Copy and paste relevant sections from the manuscript (include quotes in quotation marks "like this" to indicate direct quotes from your manuscript), or elaborate on this item by providing additional information not in the ms, or briefly explain why the item is not applicable/relevant for your study

"This research was an ancillary study of the multicentre, randomized SENTIPAT trial [26]. We took advantage of the trial to analyse patients' satisfaction with their hospital stay."
"Consecutive inpatients with Internet access at home were eligible for inclusion." ...
"Inpatients were enrolled the day of hospital discharge by a clinical research technician of the trial. At this moment, patients were informed about study. Eligible patients not opposed to participate in

3b) Important changes to methods after trial commencement (such as eligibility criteria), with reasons

Does your paper address CONSORT subitem 3b? *

Copy and paste relevant sections from the manuscript (include quotes in quotation marks "like this" to indicate direct quotes from your manuscript), or elaborate on this item by providing additional information not in the ms, or briefly explain why the item is not applicable/relevant for your study

not applicable, no change

3b-i) Bug fixes, Downtimes, Content Changes

Bug fixes, Downtimes, Content Changes: ehealth systems are often dynamic systems. A description of changes to methods therefore also includes important changes made on the intervention or comparator during the trial (e.g., major bug fixes or changes in the functionality or content) (5-iii) and other "unexpected events" that may have influenced study design such as staff changes, system failures/downtimes, etc. [2].

	1	2	3	4	5	
subitem not at all important	○	○	○	○	○	essential

Does your paper address subitem 3b-i?

Copy and paste relevant sections from the manuscript (include quotes in quotation marks "like this" to indicate direct quotes from your manuscript), or elaborate on this item by providing additional information not in the ms, or briefly explain why the item is not applicable/relevant for your study

not applicable, no change

4a) Eligibility criteria for participants

Does your paper address CONSORT subitem 4a? *

Copy and paste relevant sections from the manuscript (include quotes in quotation marks "like this" to indicate direct quotes from your manuscript), or elaborate on this item by providing additional information not in the ms, or briefly explain why the item is not applicable/relevant for your study

"Consecutive inpatients with Internet access at home were eligible for inclusion. Inclusion criteria also required inpatients who were not cognitively impaired and did not have a behavioural disorder, who spoke and wrote French, and were returning home after an acute care hospitalization, regardless of the type of stay–standard hospitalization (scheduled or not) on weekdays only (maximum Monday to Friday or any combination thereof) or outpatient hospitalization (1 day)."

4a-i) Computer / Internet literacy

Computer / Internet literacy is often an implicit "de facto" eligibility criterion - this should be explicitly clarified.

	1	2	3	4	5	
subitem not at all important	○	○	○	○	○	essential

Does your paper address subitem 4a-i?

Copy and paste relevant sections from the manuscript (include quotes in quotation marks "like this" to indicate direct quotes from your manuscript), or elaborate on this item by providing additional information not in the ms, or briefly explain why the item is not applicable/relevant for your study

"Consecutive inpatients with Internet access at home were eligible for inclusion. Inclusion criteria also required inpatients who were not cognitively impaired and did not have a behavioural disorder, who spoke and wrote French, and were returning home after an acute care hospitalization, regardless of the type of stay–standard hospitalization (scheduled or not) on weekdays only (maximum Monday to Friday or any combination thereof) or outpatient

4a-ii) Open vs. closed, web-based vs. face-to-face assessments:

Open vs. closed, web-based vs. face-to-face assessments: Mention how participants were recruited (online vs. offline), e.g., from an open access website or from a clinic, and clarify if this was a purely web-based trial, or there were face-to-face components (as part of the intervention or for assessment), i.e., to what degree got the study team to know the participant. In online-only trials, clarify if participants were quasi-anonymous and whether having multiple identities was possible or whether technical or logistical measures (e.g., cookies, email confirmation, phone calls) were used to detect/prevent these.

	1	2	3	4	5	
subitem not at all important	○	○	○	○	○	essential

Does your paper address subitem 4a-ii? *

Copy and paste relevant sections from the manuscript (include quotes in quotation marks "like this" to indicate direct quotes from your manuscript), or elaborate on this item by providing additional information not in the ms, or briefly explain why the item is not applicable/relevant for your study

“Inpatients were enrolled the day of hospital discharge by a clinical research technician of the trial.”
“Eligible patients not opposed to participate in the study patients were randomized into two parallel groups, Internet or Telephone follow-up”
“For the patients that had been randomized in the Telephone group, the I-Satis questionnaire was administered during a telephone interview with a clinical research technician”
”For the patients that had been randomized in the Internet group, the same questionnaire was available on the dedicated website on

4a-iii) Information giving during recruitment

Information given during recruitment. Specify how participants were briefed for recruitment and in the informed consent procedures (e.g., publish the informed consent documentation as appendix, see also item X26), as this information may have an effect on user self-selection, user expectation and may also bias results.

	1	2	3	4	5	
subitem not at all important	○	○	○	○	○	essential

Does your paper address subitem 4a-iii?

Copy and paste relevant sections from the manuscript (include quotes in quotation marks "like this" to indicate direct quotes from your manuscript), or elaborate on this item by providing additional information not in the ms, or briefly explain why the item is not applicable/relevant for your study

"volunteer patients"
"The study was conducted in accordance with French regulation on ethics requirements in biomedical research (see approvals of regulation institutions at the end of the document). Inpatients were enrolled the day of hospital discharge by a clinical research technician of the trial. At this moment, patients were informed about study. Eligible patients not opposed to participate in the study were randomized into two parallel groups, Internet or Telephone follow-up (inherently resulting in an open label trial), at a ratio 1:1, and the technician collected baseline variables. Permutation block randomization was stratified by service. The computer generated list of permutation was established by a statistician independent from the study. Based on a centralized randomization that allocated either Internet or Telephone through a website, patients were randomized the day of hospital discharge by a clinical research

4b) Settings and locations where the data were collected

Does your paper address CONSORT subitem 4b? *

Copy and paste relevant sections from the manuscript (include quotes in quotation marks "like this" to indicate direct quotes from your manuscript), or elaborate on this item by providing additional information not in the ms, or briefly explain why the item is not applicable/relevant for your study

"The SENTIPAT multicentre (five adult acute care units in a Parisian teaching hospital participated voluntarily: departments of Digestive and General Surgery, Gastroenterology, Hepatology, Infectious Diseases and Tropical Medicine, and Internal Medicine), randomized trial focused on the evolution of patients' health on returning home post hospitalization (follow-up duration of six

4b-i) Report if outcomes were (self-)assessed through online questionnaires

Clearly report if outcomes were (self-)assessed through online questionnaires (as common in web-based trials) or otherwise.

	1	2	3	4	5	
subitem not at all important	○	○	○	○	○	essential

Does your paper address subitem 4b-i? *

Copy and paste relevant sections from the manuscript (include quotes in quotation marks "like this" to indicate direct quotes from your manuscript), or elaborate on this item by providing additional information not in the ms, or briefly explain why the item is not applicable/relevant for your study

"Questionnaire Administration
All patients were informed that their opinions were kept anonymous. For the patients that had been randomized in the Telephone group, the I-Satis questionnaire was administered during a telephone interview with a clinical research technician 7 days after discharge (the appointment was scheduled the day of discharge), with a maximum of 3 attempts to contact them. For the patients that had been randomized in the Internet group, the same questionnaire was available on the dedicated website on the day of discharge (D0) and was completed directly online by the patient, who had been given oral and written instructions (information sheet) to connect for the first time 7 days post discharge. "Reminders" were sent by e-mail once weekly for 6 weeks after discharge to potential

4b-ii) Report how institutional affiliations are displayed

Report how institutional affiliations are displayed to potential participants [on ehealth media], as affiliations with prestigious hospitals or universities may affect volunteer rates, use, and reactions with regards to an intervention.(Not a required item – describe only if this may bias results)

	1	2	3	4	5	
subitem not at all important	○	○	○	○	○	essential

Does your paper address subitem 4b-ii?

Copy and paste relevant sections from the manuscript (include quotes in quotation marks "like this" to indicate direct quotes from your manuscript), or elaborate on this item by providing additional information not in the ms, or briefly explain why the item is not applicable/relevant for your study

in the letter of information given to potential participants:
"Madame, Monsieur,
Nous vous proposons de participer à une étude de l’Assistance Publique-Hôpitaux de Paris, sur votre prise en charge médicale à la sortie de l’hôpital et sur votre satisfaction des soins qui vous ont été procurés pendant le séjour hospitalier et après la sortie. "

5) The interventions for each group with sufficient details to allow replication, including how and when they were actually administered

5-i) Mention names, credential, affiliations of the developers, sponsors, and owners

Mention names, credential, affiliations of the developers, sponsors, and owners [6] (if authors/evaluators are owners or developer of the software, this needs to be declared in a “Conflict of interest” section or mentioned elsewhere in the manuscript).

	1	2	3	4	5	
subitem not at all important	○	○	○	○	○	essential

Does your paper address subitem 5-i?

Copy and paste relevant sections from the manuscript (include quotes in quotation marks "like this" to indicate direct quotes from your manuscript), or elaborate on this item by providing additional information not in the ms, or briefly explain why the item is not applicable/relevant for your study

"ACKNOWLEDGEMENTS
The SENTIPAT study group:
1. Scientific Committee: Fabrice Carrat, Bérengère Couturier, Gilles Hejblum, Morgane Le Bail, Alain-Jacques Valleron, and the below-mentioned heads and physicians within the poles and units in which patients were recruited.

Personnel of the study group within the poles and units concerned:
2. Heads: Marc Beaussier, Jean-Paul Cabane, Olivier Chazouillères, Jacques Cosnes, Jean-Claude Dussaule, Pierre-Marie Girard, Emmanuel Tiret and Dominique Pateron;
3. Additional corresponding physicians: Laurent Beaugerie, Laurence Fardet, Laurent Fonquernie, François Paye, Laure Surgers.
4. Nursing supervisors: Françoise Cuiller, Catherine Esnouf, Hélène Haure, Valérie Garnier, Josselin Mehal-Birba, Nelly Sallée, and Sylvie Wagener.

The authors are indebted to the technical team of the study:
The clinical research technicians: Élodie Belladame, Azéline Chevance, Magali Girard and Laurence Nicole, who included patients, collected baseline data and interviewed the patients followed-up by telephone.
The software staff, especially Pauline Raballand, but also Frédéric Chau and Frédéric Fotré, who created and maintained the trial's dedicated website;

We thank all the medical and nursing and administrative staff of the General and Digestive Surgery (including Ambulatory Surgery), Gastroenterology, Hepatology, Infectious Diseases and Internal Medicine departments of Hôpital Saint-Antoine;
We thank all patients who participated in the study.
We thank Isabelle Mounier-Emeury for helpful discussions on the calculations of I-Satis scores.

The Assistance Publique–Hôpitaux de Paris (Département de la Recherche Clinique et du Développement) was the trial sponsor.
The SENTIPAT study was funded by grant AOM09213 K081204 from Programme Hospitalier de Recherche Clinique 2009 (Ministère

5-ii) Describe the history/development process

Describe the history/development process of the application and previous formative evaluations (e.g., focus groups, usability testing), as these will have an impact on adoption/use rates and help with interpreting results.

	1	2	3	4	5	
subitem not at all important	○	○	○	○	○	essential

Does your paper address subitem 5-ii?

Copy and paste relevant sections from the manuscript (include quotes in quotation marks "like this" to indicate direct quotes from your manuscript), or elaborate on this item by providing additional information not in the ms, or briefly explain why the item is not applicable/relevant for your study

Not applicable : dedicated web site developed for the SENTIPAT trial

5-iii) Revisions and updating

Revisions and updating. Clearly mention the date and/or version number of the application/intervention (and comparator, if applicable) evaluated, or describe whether the intervention underwent major changes during the evaluation process, or whether the development and/or content was "frozen" during the trial. Describe dynamic components such as news feeds or changing content which may have an impact on the replicability of the intervention (for unexpected events see item 3b).

	1	2	3	4	5	
subitem not at all important	○	○	○	○	○	essential

Does your paper address subitem 5-iii?

Copy and paste relevant sections from the manuscript (include quotes in quotation marks "like this" to indicate direct quotes from your manuscript), or elaborate on this item by providing additional information not in the ms, or briefly explain why the item is not applicable/relevant for your study

not applicable: no revisions and updating

5-iv) Quality assurance methods

Provide information on quality assurance methods to ensure accuracy and quality of information provided [1], if applicable.

	1	2	3	4	5	
subitem not at all important	○	○	○	○	○	essential

Does your paper address subitem 5-iv?

Copy and paste relevant sections from the manuscript (include quotes in quotation marks "like this" to indicate direct quotes from your manuscript), or elaborate on this item by providing additional information not in the ms, or briefly explain why the item is not applicable/relevant for your study

not applicable

5-v) Ensure replicability by publishing the source code, and/or providing screenshots/screen-capture video, and/or providing flowcharts of the algorithms used

Ensure replicability by publishing the source code, and/or providing screenshots/screen-capture video, and/or providing flowcharts of the algorithms used. Replicability (i.e., other researchers should in principle be able to replicate the study) is a hallmark of scientific reporting.

	1	2	3	4	5	
subitem not at all important	○	○	○	○	○	essential

Does your paper address subitem 5-v?

Copy and paste relevant sections from the manuscript (include quotes in quotation marks "like this" to indicate direct quotes from your manuscript), or elaborate on this item by providing additional information not in the ms, or briefly explain why the item is not applicable/relevant for your study

not appicable

5-vi) Digital preservation

Digital preservation: Provide the URL of the application, but as the intervention is likely to change or disappear over the course of the years; also make sure the intervention is archived (Internet Archive, webcitation.org, and/or publishing the source code or screenshots/videos alongside the article). As pages behind login screens cannot be archived, consider creating demo pages which are accessible without login.

	1	2	3	4	5	
subitem not at all important	○	○	○	○	○	essential

Does your paper address subitem 5-vi?

Copy and paste relevant sections from the manuscript (include quotes in quotation marks "like this" to indicate direct quotes from your manuscript), or elaborate on this item by providing additional information not in the ms, or briefly explain why the item is not applicable/relevant for your study

not applicable

5-vii) Access

Access: Describe how participants accessed the application, in what setting/context, if they had to pay (or were paid) or not, whether they had to be a member of specific group. If known, describe how participants obtained “access to the platform and Internet” [1]. To ensure access for editors/reviewers/readers, consider to provide a “backdoor” login account or demo mode for reviewers/readers to explore the application (also important for archiving purposes, see vi).

	1	2	3	4	5	
subitem not at all important	○	○	○	○	○	essential

Does your paper address subitem 5-vii? *

Copy and paste relevant sections from the manuscript (include quotes in quotation marks "like this" to indicate direct quotes from your manuscript), or elaborate on this item by providing additional information not in the ms, or briefly explain why the item is not applicable/relevant for your study

"Questionnaire Administration
All patients were informed that their opinions were kept anonymous. For the patients that had been randomized in the Telephone group, the I-Satis questionnaire was administered during a telephone interview with a clinical research technician 7 days after discharge (the appointment was scheduled the day of discharge), with a maximum of 3 attempts to contact them. For the patients that had been randomized in the Internet group, the same questionnaire was available on the dedicated website on the day of discharge (D0) and was completed directly online by the patient, who had been given oral and written instructions (information sheet) to connect for the first time 7 days post discharge. “Reminders” were sent by e-mail once weekly for 6 weeks after discharge to potential

5-viii) Mode of delivery, features/functionalities/components of the intervention and

comparator, and the theoretical framework

Describe mode of delivery, features/functionalities/components of the intervention and comparator, and the theoretical framework [6] used to design them (instructional strategy [1], behaviour change techniques, persuasive features, etc., see e.g., [7, 8] for terminology). This includes an in-depth description of the content (including where it is coming from and who developed it) [1],” whether [and how] it is tailored to individual circumstances and allows users to track their progress and receive feedback” [6]. This also includes a description of communication delivery channels and – if computer-mediated communication is a component – whether communication was synchronous or asynchronous [6]. It also includes information on presentation strategies [1], including page design principles, average amount of text on pages, presence of hyperlinks to other resources, etc. [1].

	1	2	3	4	5	
subitem not at all important	○	○	○	○	○	essential

Does your paper address subitem 5-viii? *

Copy and paste relevant sections from the manuscript (include quotes in quotation marks "like this" to indicate direct quotes from your manuscript), or elaborate on this item by providing additional information not in the ms, or briefly explain why the item is not applicable/relevant for your study

Questionnaire Administration
All patients were informed that their opinions were kept anonymous. For the patients that had been randomized in the Telephone group, the I-Satis questionnaire was administered during a telephone interview with a clinical research technician 7 days after discharge (the appointment was scheduled the day of discharge), with a maximum of 3 attempts to contact them. For the patients that had been randomized in the Internet group, the same questionnaire was available on the dedicated website on the day of discharge (D0) and was completed directly online by the patient, who had been given oral and written instructions (information sheet) to connect for the first time 7 days post discharge. “Reminders” were sent by e-mail once weekly for 6 weeks after discharge to potential

5-ix) Describe use parameters

Describe use parameters (e.g., intended “doses” and optimal timing for use). Clarify what instructions or recommendations were given to the user, e.g., regarding timing, frequency, heaviness of use, if any, or was the intervention used ad libitum.

	1	2	3	4	5	
subitem not at all important	○	○	○	○	○	essential

Does your paper address subitem 5-ix?

Copy and paste relevant sections from the manuscript (include quotes in quotation marks "like this" to indicate direct quotes from your manuscript), or elaborate on this item by providing additional information not in the ms, or briefly explain why the item is not applicable/relevant for your study

Questionnaire Administration
All patients were informed that their opinions were kept anonymous. For the patients that had been randomized in the Telephone group, the I-Satis questionnaire was administered during a telephone interview with a clinical research technician 7 days after discharge (the appointment was scheduled the day of discharge), with a maximum of 3 attempts to contact them. For the patients that had been randomized in the Internet group, the same questionnaire was available on the dedicated website on the day of discharge (D0) and was completed directly online by the patient, who had been given oral and written instructions (information sheet) to connect for the first time 7 days post discharge. “Reminders” were sent by e-mail once weekly for 6 weeks after discharge to potential [illegible]

5-x) Clarify the level of human involvement

Clarify the level of human involvement (care providers or health professionals, also technical assistance) in the e-intervention or as co-intervention (detail number and expertise of professionals involved, if any, as well as “type of assistance offered, the timing and frequency of the support, how it is initiated, and the medium by which the assistance is delivered”. It may be necessary to distinguish between the level of human involvement required for the trial, and the level of human involvement required for a routine application outside of a RCT setting (discuss under item 21 – generalizability).

	1	2	3	4	5	
subitem not at all important	○	○	○	○	○	essential

Does your paper address subitem 5-x?

Copy and paste relevant sections from the manuscript (include quotes in quotation marks "like this" to indicate direct quotes from your manuscript), or elaborate on this item by providing additional information not in the ms, or briefly explain why the item is not applicable/relevant for your study

Questionnaire Administration
All patients were informed that their opinions were kept anonymous. For the patients that had been randomized in the Telephone group, the I-Satis questionnaire was administered during a telephone interview with a clinical research technician 7 days after discharge (the appointment was scheduled the day of discharge), with a maximum of 3 attempts to contact them. For the patients that had been randomized in the Internet group, the same questionnaire was available on the dedicated website on the day of discharge (D0) and was completed directly online by the patient, who had been given oral and written instructions (information sheet) to connect for the first time 7 days post discharge. “Reminders” were sent by e-mail once weekly for 6 weeks after discharge to potential [illegible]

5-xi) Report any prompts/reminders used

Report any prompts/reminders used: Clarify if there were prompts (letters, emails, phone calls, SMS) to use the application, what triggered them, frequency etc. It may be necessary to distinguish between the level of prompts/reminders required for the trial, and the level of prompts/reminders for a routine application outside of a RCT setting (discuss under item 21 – generalizability).

	1	2	3	4	5	
subitem not at all important	○	○	○	○	○	essential

Does your paper address subitem 5-xi? *

Copy and paste relevant sections from the manuscript (include quotes in quotation marks "like this" to indicate direct quotes from your manuscript), or elaborate on this item by providing additional information not in the ms, or briefly explain why the item is not applicable/relevant for your study

Questionnaire Administration
All patients were informed that their opinions were kept anonymous. For the patients that had been randomized in the Telephone group, the I-Satis questionnaire was administered during a telephone interview with a clinical research technician 7 days after discharge (the appointment was scheduled the day of discharge), with a maximum of 3 attempts to contact them. For the patients that had been randomized in the Internet group, the same questionnaire was available on the dedicated website on the day of discharge (D0) and was completed directly online by the patient, who had been given oral and written instructions (information sheet) to connect for the first time 7 days post discharge. “Reminders” were sent by e-mail once weekly for 6 weeks after discharge to potential

5-xii) Describe any co-interventions (incl. training/support)

Describe any co-interventions (incl. training/support): Clearly state any interventions that are provided in addition to the targeted eHealth intervention, as ehealth intervention may not be designed as stand-alone intervention. This includes training sessions and support [1]. It may be necessary to distinguish between the level of training required for the trial, and the level of training for a routine application outside of a RCT setting (discuss under item 21 – generalizability.

	1	2	3	4	5	
subitem not at all important	○	○	○	○	○	essential

Does your paper address subitem 5-xii? *

Copy and paste relevant sections from the manuscript (include quotes in quotation marks "like this" to indicate direct quotes from your manuscript), or elaborate on this item by providing additional information not in the ms, or briefly explain why the item is not applicable/relevant for your study

not applicable

6a) Completely defined pre-specified primary and secondary outcome measures, including how and when they were assessed

Does your paper address CONSORT subitem 6a? *

Copy and paste relevant sections from the manuscript (include quotes in quotation marks "like this" to indicate direct quotes from your manuscript), or elaborate on this item by providing additional information not in the ms, or briefly explain why the item is not applicable/relevant for

your study

Not strictly applicable for this ancillary study.
"“The questionnaires were analysed according to the French national recommendations of the Direction Générale de l'Offre de Soins.”
“The scores of the dimensions “global care”, “information to patient”, “communication with healthcare providers” “behaviour of healthcare providers”, “hospital room convenience”, and “hospital catering” were calculated if at least three, three, three, three, two, and two items composing the dimension were answered, respectively. The global score was calculated whenever every dimension score was calculated.”
...
“Internal consistency of questionnaires was measured by calculating Cronbach’s alpha.”
....
“Dimensions’ scores were calculated for each patient as the mean of the corresponding dimensions’ items and global score was the mean of all answered items of the questionnaire”
...
"Confidence intervals (CI) were obtained by bootstrap.
Standardized Cohen’s d-type effect size was measured between scores of the two groups [29].
Comparisons between the Internet and Telephone group in terms of Cronbach’s alpha coefficients as well as in terms of satisfaction scores (including dimensions’ scores) were made using a permutation test [30], with the null hypothesis distribution (distribution of the difference between the two groups under the hypothesis of no difference) generated through 1,000,000 shuffled data sets. A P value ≤ .05 defined significance of comparisons.
Missing data were handled as follows: First, nonresponding patients were excluded from score analyses. Patients for which less than 16 items were completed were also excluded from score analyses (i.e. handled as nonresponders in the analyses) Second, the scores issued from the remaining partially completed questionnaires were calculated as above-mentioned (see subsection score construction).

6a-i) Online questionnaires: describe if they were validated for online use and apply CHERRIES items to describe how the questionnaires were designed/deployed

If outcomes were obtained through online questionnaires, describe if they were validated for online use and apply CHERRIES items to describe how the questionnaires were designed/deployed [9].

	1	2	3	4	5	
subitem not at all important	○	○	○	○	○	essential

Does your paper address subitem 6a-i?

Copy and paste relevant sections from manuscript text

not applicable: The French Ministry of Helath has made The I-Satis questionnaire as a kind of "mandatory" tool. French authorities require the analysis of 120 patients in each French medical centre each year, for assessing patient satisfaction in the corresponding

6a-ii) Describe whether and how “use” (including intensity of use/dosage) was

defined/measured/monitored

Describe whether and how “use” (including intensity of use/dosage) was defined/measured /monitored (logins, logfile analysis, etc.). Use/adoption metrics are important process outcomes that should be reported in any ehealth trial.

	1	2	3	4	5	
subitem not at all important	○	○	○	○	○	essential

Does your paper address subitem 6a-ii?

Copy and paste relevant sections from manuscript text

not applicable

6a-iii) Describe whether, how, and when qualitative feedback from participants was obtained

Describe whether, how, and when qualitative feedback from participants was obtained (e.g., through emails, feedback forms, interviews, focus groups).

	1	2	3	4	5	
subitem not at all important	○	○	○	○	○	essential

Does your paper address subitem 6a-iii?

Copy and paste relevant sections from manuscript text

not applicable (n.b. getting feedback from responders to a questionnaire on satisfaction would be a kind of vicious cercle)

6b) Any changes to trial outcomes after the trial commenced, with reasons

Does your paper address CONSORT subitem 6b? *

Copy and paste relevant sections from the manuscript (include quotes in quotation marks "like this" to indicate direct quotes from your manuscript), or elaborate on this item by providing additional information not in the ms, or briefly explain why the item is not applicable/relevant for your study

not applicable

7a) How sample size was determined

NPT: When applicable, details of whether and how the clustering by care provides or centers was addressed

7a-i) Describe whether and how expected attrition was taken into account when calculating the sample size

Describe whether and how expected attrition was taken into account when calculating the sample size.

	1	2	3	4	5	
subitem not at all important	○	○	○	○	○	essential

Does your paper address subitem 7a-i?

Copy and paste relevant sections from manuscript title (include quotes in quotation marks "like this" to indicate direct quotes from your manuscript), or elaborate on this item by providing additional information not in the ms, or briefly explain why the item is not applicable/relevant for your study

not applicable for this ancillary study.
See sample size calculation for the main study at
https://clinicaltrials.gov/ct2/show/record/NCT01769261

7b) When applicable, explanation of any interim analyses and stopping guidelines

Does your paper address CONSORT subitem 7b? *

Copy and paste relevant sections from the manuscript (include quotes in quotation marks "like this" to indicate direct quotes from your manuscript), or elaborate on this item by providing additional information not in the ms, or briefly explain why the item is not applicable/relevant for your study

No intermediate analysis was planned

8a) Method used to generate the random allocation sequence

NPT: When applicable, how care providers were allocated to each trial group

Does your paper address CONSORT subitem 8a? *

Copy and paste relevant sections from the manuscript (include quotes in quotation marks "like this" to indicate direct quotes from your manuscript), or elaborate on this item by providing additional information not in the ms, or briefly explain why the item is not applicable/relevant for your study

Eligible patients not opposed to participate in the study were randomized into two parallel groups, Internet or Telephone follow-up (inherently resulting in an open label trial), at a ratio 1:1, and the technician collected baseline variables. Permutation block randomization was stratified by service. The computer generated list of permutation was established by a statistician independent from the study. Based on a centralized randomization that allocated either Internet or Telephone through a website, patients were randomized the day of hospital discharge by a clinical research technician, and patient was informed and discharged with

8b) Type of randomisation; details of any restriction (such as blocking and block size)

Does your paper address CONSORT subitem 8b? *

Copy and paste relevant sections from the manuscript (include quotes in quotation marks "like this" to indicate direct quotes from your manuscript), or elaborate on this item by providing additional information not in the ms, or briefly explain why the item is not applicable/relevant for your study

“ Permutation block randomization was stratified by service.”

9) Mechanism used to implement the random allocation sequence (such as sequentially numbered containers), describing any steps taken to conceal the sequence until interventions were assigned

Does your paper address CONSORT subitem 9? *

Copy and paste relevant sections from the manuscript (include quotes in quotation marks "like this" to indicate direct quotes from your manuscript), or elaborate on this item by providing additional information not in the ms, or briefly explain why the item is not applicable/relevant for your study

“Based on a centralized randomization that allocated either Internet or Telephone through a website, patients were randomized the day of hospital discharge”

10) Who generated the random allocation sequence, who enrolled participants, and who assigned participants to interventions

Does your paper address CONSORT subitem 10? *

Copy and paste relevant sections from the manuscript (include quotes in quotation marks "like this" to indicate direct quotes from your manuscript), or elaborate on this item by providing additional information not in the ms, or briefly explain why the item is not applicable/relevant for your study

“Inpatients were enrolled the day of hospital discharge by a clinical research technician of the trial.”
“The computer generated list of permutation was established by a statistician independent from the study.”
“Based on a centralized randomization that allocated either Internet or Telephone through a website, patients were randomized the day of hospital discharge by a clinical research technician.”

11a) If done, who was blinded after assignment to interventions (for example, participants, care providers, those assessing outcomes) and how

NPT: Whether or not administering co-interventions were blinded to group assignment

11a-i) Specify who was blinded, and who wasn’t

Specify who was blinded, and who wasn’t. Usually, in web-based trials it is not possible to blind the

participants [1, 3] (this should be clearly acknowledged), but it may be possible to blind outcome assessors, those doing data analysis or those administering co-interventions (if any).

	1	2	3	4	5	
subitem not at all important	○	○	○	○	○	essential

Does your paper address subitem 11a-i? *

Copy and paste relevant sections from the manuscript (include quotes in quotation marks "like this" to indicate direct quotes from your manuscript), or elaborate on this item by providing additional information not in the ms, or briefly explain why the item is not applicable/relevant for your study

“inherently resulting in an open label trial”

11a-ii) Discuss e.g., whether participants knew which intervention was the “intervention of interest” and which one was the “comparator”

Informed consent procedures (4a-ii) can create biases and certain expectations - discuss e.g., whether participants knew which intervention was the “intervention of interest” and which one was the “comparator”.

	1	2	3	4	5	
subitem not at all important	○	○	○	○	○	essential

Does your paper address subitem 11a-ii?

Copy and paste relevant sections from the manuscript (include quotes in quotation marks "like this" to indicate direct quotes from your manuscript), or elaborate on this item by providing additional information not in the ms, or briefly explain why the item is not applicable/relevant for your study

“patient was informed and discharged with documents explaining corresponding questionnaire administration”

11b) If relevant, description of the similarity of interventions

(this item is usually not relevant for ehealth trials as it refers to similarity of a placebo or sham intervention to a active medication/intervention)

Does your paper address CONSORT subitem 11b? *

Copy and paste relevant sections from the manuscript (include quotes in quotation marks "like this" to indicate direct quotes from your manuscript), or elaborate on this item by providing additional information not in the ms, or briefly explain why the item is not applicable/relevant for your study

not applicable

12a) Statistical methods used to compare groups for

primary and secondary outcomes

NPT: When applicable, details of whether and how the clustering by care providers or centers was addressed

Does your paper address CONSORT subitem 12a? *

Copy and paste relevant sections from the manuscript (include quotes in quotation marks "like this" to indicate direct quotes from your manuscript), or elaborate on this item by providing additional information not in the ms, or briefly explain why the item is not applicable/relevant for your study

Statistical analysis
The participation rates observed in the Internet and Telephone groups were compared using Fisher exact test, as well as the proportions of non-relevancy answers observed in these two groups. The delays of questionnaire completion observed in the Internet and Telephone groups were compared using Wilcoxon-Mann-Whitney test. Internal consistency of questionnaires was measured by calculating Cronbach's alpha [28], taking into account every score that could be calculated according to the rules above-mentioned. An alpha coefficient value greater than 0.7 was considered as satisfactory. Dimensions' scores were calculated for each patient as the mean of the corresponding dimensions' items and global score was the mean of all answered items of the questionnaire. Confidence intervals (CI) were obtained by bootstrap. Standardized Cohen's d-type effect size was measured between scores of the two groups [29].
Comparisons between the Internet and Telephone group in terms of Cronbach's alpha coefficients as well as in terms of satisfaction scores (including dimensions' scores) were made using a permutation test [30], with the null hypothesis distribution (distribution of the difference between the two groups under the hypothesis of no difference) generated through 1,000,000 shuffled data sets. A P value ≤ .05 defined significance of comparisons.
Missing data were handled as follows: First, nonresponding patients were excluded from score analyses. Patients for which less than 16 items were completed were also excluded from score analyses (i.e. handled as nonresponders in the analyses) Second, the scores issued from the remaining partially completed questionnaires were calculated as above-mentioned (see subsection score construction).

12a-i) Imputation techniques to deal with attrition / missing values

Imputation techniques to deal with attrition / missing values: Not all participants will use the intervention/comparator as intended and attrition is typically high in ehealth trials. Specify how participants who did not use the application or dropped out from the trial were treated in the statistical analysis (a complete case analysis is strongly discouraged, and simple imputation techniques such as LOCF may also be problematic [4]).

	1	2	3	4	5	
subitem not at all important	○	○	○	○	○	essential

Does your paper address subitem 12a-i? *

Copy and paste relevant sections from the manuscript (include quotes in quotation marks "like this" to indicate direct quotes from your manuscript), or elaborate on this item by providing additional information not in the ms, or briefly explain why the item is not applicable/relevant for your study

"Rates 0, 6, 7, 8, 9 and 10 were handled into analyses as a missing value."
...
"Missing data were handled as follows: First, nonresponding patients were excluded from score analyses. Patients for which less than 16 items were completed were also excluded from score analyses (i.e. handled as nonresponders in the analyses) Second, the scores issued from the remaining partially completed questionnaires were calculated as above-mentioned (see

12b) Methods for additional analyses, such as subgroup analyses and adjusted analyses

Does your paper address CONSORT subitem 12b? *

Copy and paste relevant sections from the manuscript (include quotes in quotation marks "like this" to indicate direct quotes from your manuscript), or elaborate on this item by providing additional information not in the ms, or briefly explain why the item is not applicable/relevant for your study

not applicable

X26) REB/IRB Approval and Ethical Considerations [recommended as subheading under "Methods"] (not a CONSORT item)

X26-i) Comment on ethics committee approval

	1	2	3	4	5	
subitem not at all important	○	○	○	○	○	essential

Does your paper address subitem X26-i?

Copy and paste relevant sections from the manuscript (include quotes in quotation marks "like this" to indicate direct quotes from your manuscript), or elaborate on this item by providing additional information not in the ms, or briefly explain why the item is not applicable/relevant for your study

ETHIC AND LEGAL APPROVALS
The SENTIPAT study was approved by the Comité de Protection des Personnes Ile de France IX (decision CPP-IDF IX 12-014, June 12, 2012), by the Comité Consultatif sur le Traitement de l'Information en matière de Recherche dans le domaine de la Santé (Decision 12.365, June 20, 2012), and by the Commission Nationale de l'Informatique et des Libertés (Decision DR-2012-582, December 12, 2012).

x26-ii) Outline informed consent procedures

Outline informed consent procedures e.g., if consent was obtained offline or online (how? Checkbox, etc.?), and what information was provided (see 4a-ii). See [6] for some items to be included in informed consent documents.

1 2 3 4 5

subitem not at all important ○ ○ ○ ○ ○ essential

Does your paper address subitem X26-ii?

Copy and paste relevant sections from the manuscript (include quotes in quotation marks "like this" to indicate direct quotes from your manuscript), or elaborate on this item by providing additional information not in the ms, or briefly explain why the item is not applicable/relevant for your study

"The study was conducted in accordance with French regulation on ethics requirements in biomedical research (see approvals of regulation institutions at the end of the document). "
...
"Eligible patients not opposed to participate in the study patients were randomized"

X26-iii) Safety and security procedures

Safety and security procedures, incl. privacy considerations, and any steps taken to reduce the likelihood or detection of harm (e.g., education and training, availability of a hotline)

1 2 3 4 5

subitem not at all important ○ ○ ○ ○ ○ essential

Does your paper address subitem X26-iii?

Copy and paste relevant sections from the manuscript (include quotes in quotation marks "like this" to indicate direct quotes from your manuscript), or elaborate on this item by providing additional information not in the ms, or briefly explain why the item is not applicable/relevant for your study

Not applicable for the ancillary study.
For the Sentipat randomized trial, see https://clinicaltrials.gov /ct2/show/record/NCT01769261

RESULTS

13a) For each group, the numbers of participants who were randomly assigned, received intended treatment, and were analysed for the primary outcome

NPT: The number of care providers or centers performing the intervention in each group and the number of patients treated by each care provider in each center

Does your paper address CONSORT subitem 13a? *

Copy and paste relevant sections from the manuscript (include quotes in quotation marks "like this" to indicate direct quotes from your manuscript), or elaborate on this item by providing additional information not in the ms, or briefly explain why the item is not applicable/relevant for

your study

Figure 1

“Between February 25, 2013 and September 8, 2014, we managed to enroll 1680 eligible patients (840 and 840 were randomized in the Internet and Telephone group, respectively) and not opposed to participating in the trial. Since the outpatients that were included in the SENTIPAT study do not fulfil the minimum LOS required for

13b) For each group, losses and exclusions after randomisation, together with reasons

Does your paper address CONSORT subitem 13b? (NOTE: Preferably, this is shown in a CONSORT flow diagram) *

Copy and paste relevant sections from the manuscript (include quotes in quotation marks "like this" to indicate direct quotes from your manuscript), or elaborate on this item by providing additional information not in the ms, or briefly explain why the item is not applicable/relevant for your study

Figure 1
Missing data in responders concerned 10 patients of the Internet group: answer to question 13 (satisfaction about pain management) was missing in 2 responders, answer to question 20 (satisfaction about the answers of the surgeon about patient’s questions on surgery) was missing in 3 responders, and answer to both questions was missing in 5 responders. In addition, there were 13 (8%), 95 (62%), 43 (28%), and 3 (2%) responders in the Internet group with 0, 1 to 5, 6 to 10, and more than 10 answers for which the answer code corresponded to non-relevancy or refusal (further handled as a missing value in the analyses, see section methods), respectively, while the corresponding responders observed in the Telephone group were 3 (1%), 124 (36%), 200 (58%), and 15 (5%), respectively. Internet responders provided an answer code

13b-i) Attrition diagram

Strongly recommended: An attrition diagram (e.g., proportion of participants still logging in or using the intervention/comparator in each group plotted over time, similar to a survival curve) or other figures or tables demonstrating usage/dose/engagement.

	1	2	3	4	5	
subitem not at all important	○	○	○	○	○	essential

Does your paper address subitem 13b-i?

Copy and paste relevant sections from the manuscript or cite the figure number if applicable (include quotes in quotation marks "like this" to indicate direct quotes from your manuscript), or elaborate on this item by providing additional information not in the ms, or briefly explain why the item is not applicable/relevant for your study

Figure 1
Missing data in responders concerned 10 patients of the Internet group: answer to question 13 (satisfaction about pain management) was missing in 2 responders, answer to question 20 (satisfaction about the answers of the surgeon about patient's questions on surgery) was missing in 3 responders, and answer to both questions was missing in 5 responders. In addition, there were 13 (8%), 95 (62%), 43 (28%), and 3 (2%) responders in the Internet group with 0, 1 to 5, 6 to 10, and more than 10 answers for which the answer code corresponded to non-relevancy or refusal (further handled as a missing value in the analyses, see section methods), respectively, while the corresponding responders observed in the Telephone group were 3 (1%), 124 (36%), 200 (58%), and 15 (5%), respectively. Internet responders provided an answer code

14a) Dates defining the periods of recruitment and follow-up

Does your paper address CONSORT subitem 14a? *

Copy and paste relevant sections from the manuscript (include quotes in quotation marks "like this" to indicate direct quotes from your manuscript), or elaborate on this item by providing additional information not in the ms, or briefly explain why the item is not applicable/relevant for your study

"Between February 25, 2013 and September 8, 2014"

14a-i) Indicate if critical "secular events" fell into the study period

Indicate if critical "secular events" fell into the study period, e.g., significant changes in Internet resources available or "changes in computer hardware or Internet delivery resources"

	1	2	3	4	5	
subitem not at all important	○	○	○	○	○	essential

Does your paper address subitem 14a-i?

Copy and paste relevant sections from the manuscript (include quotes in quotation marks "like this" to indicate direct quotes from your manuscript), or elaborate on this item by providing additional information not in the ms, or briefly explain why the item is not applicable/relevant for your study

not applicable

14b) Why the trial ended or was stopped (early)

Does your paper address CONSORT subitem 14b? *

Copy and paste relevant sections from the manuscript (include quotes in quotation marks "like this" to indicate direct quotes from your manuscript), or elaborate on this item by providing additional information not in the ms, or briefly explain why the item is not applicable/relevant for your study

not applicable

15) A table showing baseline demographic and clinical characteristics for each group

NPT: When applicable, a description of care providers (case volume, qualification, expertise, etc.) and centers (volume) in each group

Does your paper address CONSORT subitem 15? *

Copy and paste relevant sections from the manuscript (include quotes in quotation marks "like this" to indicate direct quotes from your manuscript), or elaborate on this item by providing additional information not in the ms, or briefly explain why the item is not applicable/relevant for your study

Table 1

15-i) Report demographics associated with digital divide issues

In ehealth trials it is particularly important to report demographics associated with digital divide issues, such as age, education, gender, social-economic status, computer/Internet/ehealth literacy of the participants, if known.

	1	2	3	4	5	
subitem not at all important	○	○	○	○	○	essential

Does your paper address subitem 15-i? *

Copy and paste relevant sections from the manuscript (include quotes in quotation marks "like this" to indicate direct quotes from your manuscript), or elaborate on this item by providing additional information not in the ms, or briefly explain why the item is not applicable/relevant for your study

Table 1

16) For each group, number of participants (denominator) included in each analysis and whether the analysis was by original assigned groups

16-i) Report multiple “denominators” and provide definitions

Report multiple “denominators” and provide definitions: Report N’s (and effect sizes) “across a range of study participation [and use] thresholds” [1], e.g., N exposed, N consented, N used more than x times, N used more than y weeks, N participants “used” the intervention/comparator at specific pre-defined time points of interest (in absolute and relative numbers per group). Always clearly define “use” of the intervention.

	1	2	3	4	5	
subitem not at all important	○	○	○	○	○	essential

Does your paper address subitem 16-i? *

Copy and paste relevant sections from the manuscript (include quotes in quotation marks "like this" to indicate direct quotes from your manuscript), or elaborate on this item by providing additional information not in the ms, or briefly explain why the item is not applicable/relevant for your study

Table 2 and Table 3
...
Table 2 shows the values of Cronbach's alphas in the Internet and Telephone responders. All estimates excepted those corresponding to room convenience were >0.7. The alpha estimates observed in the Internet group were always greater than those observed in the Telephone group, the difference being statistically significant for 2 dimensions, global care and room convenience (P=.003 and P=.034, respectively), and for the global satisfaction (P=.034).
Table 3 summarizes the satisfaction scores observed in the Internet and Telephone groups. The mean global satisfaction score was 68.89 (95%CI 66.36–71.36) in the Internet group and 72.01 (95%CI 70.36–73.58) in the Telephone group. In both groups, the dimension that received the lowest score was hospital catering, with respective means of 45.77 (95%CI 42.18–49.39) and 45.70 (95%CI 43.32–48.06) in the Internet and Telephone group. Conversely, in both groups, the theme that received the highest score was behaviour of healthcare providers, with respective means of 87.49 (95%CI 85.05–89.73) and 92.14 (95%CI 90.81–93.39) in the Internet and Telephone group. There were three dimension scores significantly smaller in the Internet group than in the Telephone group: information to patients with a mean difference of -5.38 (P=.008), communication with health providers with a mean difference of -7.16 (P=.003), and behaviour of healthcare providers with a mean of -4.66 (P<.001). The global satisfaction score was significantly smaller in the Internet group, a mean difference of -7.16 (P=.003), and behaviour of healthcare providers with a mean [illegible]

16-ii) Primary analysis should be intent-to-treat

Primary analysis should be intent-to-treat, secondary analyses could include comparing only "users", with the appropriate caveats that this is no longer a randomized sample (see 18-i).

	1	2	3	4	5	
subitem not at all important	○	○	○	○	○	essential

Does your paper address subitem 16-ii?

Copy and paste relevant sections from the manuscript (include quotes in quotation marks "like this" to indicate direct quotes from your manuscript), or elaborate on this item by providing additional information not in the ms, or briefly explain why the item is not applicable/relevant for your study

intent-to-treat per se

17a) For each primary and secondary outcome, results for each group, and the estimated effect size and its precision (such as 95% confidence interval)

Does your paper address CONSORT subitem 17a? *

Copy and paste relevant sections from the manuscript (include quotes in quotation marks "like this" to indicate direct quotes from your manuscript), or elaborate on this item by providing additional information not in the ms, or briefly explain why the item is not applicable/relevant for your study

"There were 154 (39%) and 344 (88%) responders in the Internet and Telephone group, respectively (P<.001), "
...
"The alpha estimates observed in the Internet group were always greater than those observed in the Telephone group, the difference being statistically significant for 2 dimensions, global care and room convenience (P=.003 and P=.034, respectively), and for the global satisfaction (P=.034)."
"The mean global satisfaction score was 68.89 (95%CI 66.36–71.36) in the Internet group and 72.01 (95%CI 70.36–73.58) in the Telephone group. In both groups, the dimension that received the lowest score was hospital catering, with respective means of 45.77 (95%CI 42.18–49.39) and 45.70 (95%CI 43.32–48.06) in the Internet and Telephone group. Conversely, in both groups, the theme that received the highest score was behaviour of healthcare providers, with respective means of 87.49 (95%CI 85.05–89.73) and 92.14 (95%CI 90.81–93.39) in the Internet and Telephone group. There were three dimension scores significantly smaller in the Internet group than in the Telephone group: information to patients with a mean difference of -5.38 (P=.008), communication with health providers with a mean difference of -7.16 (P=.003), and behaviour of healthcare providers with a mean of -4.66 (P<.001). The global satisfaction score was significantly smaller in the Internet group, a mean difference of -7.16 (P=.003), and behaviour of healthcare providers with a mean of -4.66 (P<.001). The global

17a-i) Presentation of process outcomes such as metrics of use and intensity of use

In addition to primary/secondary (clinical) outcomes, the presentation of process outcomes such as metrics of use and intensity of use (dose, exposure) and their operational definitions is critical. This does not only refer to metrics of attrition (13-b) (often a binary variable), but also to more continuous exposure metrics such as “average session length”. These must be accompanied by a technical description how a metric like a “session” is defined (e.g., timeout after idle time) [1] (report under item 6a).

	1	2	3	4	5	
subitem not at all important	○	○	○	○	○	essential

Does your paper address subitem 17a-i?

Copy and paste relevant sections from the manuscript (include quotes in quotation marks "like this" to indicate direct quotes from your manuscript), or elaborate on this item by providing additional information not in the ms, or briefly explain why the item is not applicable/relevant for your study

"There were 154 (39%) and 344 (88%) responders in the Internet and Telephone group, respectively (P<.001), "

17b) For binary outcomes, presentation of both

absolute and relative effect sizes is recommended

Does your paper address CONSORT subitem 17b? *

Copy and paste relevant sections from the manuscript (include quotes in quotation marks "like this" to indicate direct quotes from your manuscript), or elaborate on this item by providing additional information not in the ms, or briefly explain why the item is not applicable/relevant for your study

not applicable

18) Results of any other analyses performed, including subgroup analyses and adjusted analyses, distinguishing pre-specified from exploratory

Does your paper address CONSORT subitem 18? *

Copy and paste relevant sections from the manuscript (include quotes in quotation marks "like this" to indicate direct quotes from your manuscript), or elaborate on this item by providing additional information not in the ms, or briefly explain why the item is not applicable/relevant for your study

not applicable

18-i) Subgroup analysis of comparing only users

A subgroup analysis of comparing only users is not uncommon in ehealth trials, but if done, it must be stressed that this is a self-selected sample and no longer an unbiased sample from a randomized trial (see 16-iii).

	1	2	3	4	5	
subitem not at all important	○	○	○	○	○	essential

Does your paper address subitem 18-i?

Copy and paste relevant sections from the manuscript (include quotes in quotation marks "like this" to indicate direct quotes from your manuscript), or elaborate on this item by providing additional information not in the ms, or briefly explain why the item is not applicable/relevant for your study

notapplicable

19) All important harms or unintended effects in each group

(for specific guidance see CONSORT for harms)

Does your paper address CONSORT subitem 19? *

Copy and paste relevant sections from the manuscript (include quotes in quotation marks "like this" to indicate direct quotes from your manuscript), or elaborate on this item by providing additional information not in the ms, or briefly explain why the item is not applicable/relevant for your study

not applicable

19-i) Include privacy breaches, technical problems

Include privacy breaches, technical problems. This does not only include physical “harm” to participants, but also incidents such as perceived or real privacy breaches [1], technical problems, and other unexpected/unintended incidents. “Unintended effects” also includes unintended positive effects [2].

	1	2	3	4	5	
subitem not at all important	○	○	○	○	○	essential

Does your paper address subitem 19-i?

Copy and paste relevant sections from the manuscript (include quotes in quotation marks "like this" to indicate direct quotes from your manuscript), or elaborate on this item by providing additional information not in the ms, or briefly explain why the item is not applicable/relevant for your study

not applicable

19-ii) Include qualitative feedback from participants or observations from staff/researchers

Include qualitative feedback from participants or observations from staff/researchers, if available, on strengths and shortcomings of the application, especially if they point to unintended/unexpected effects or uses. This includes (if available) reasons for why people did or did not use the application as intended by the developers.

	1	2	3	4	5	
subitem not at all important	○	○	○	○	○	essential

Does your paper address subitem 19-ii?

Copy and paste relevant sections from the manuscript (include quotes in quotation marks "like this" to indicate direct quotes from your manuscript), or elaborate on this item by providing additional information not in the ms, or briefly explain why the item is not applicable/relevant for your study

not applicable

DISCUSSION

22) Interpretation consistent with results, balancing benefits and harms, and considering other relevant evidence

NPT: In addition, take into account the choice of the comparator, lack of or partial blinding, and unequal expertise of care providers or centers in each group

22-i) Restate study questions and summarize the answers suggested by the data,

starting with primary outcomes and process outcomes (use)

Restate study questions and summarize the answers suggested by the data, starting with primary outcomes and process outcomes (use).

	1	2	3	4	5	
subitem not at all important	○	○	○	○	○	essential

Does your paper address subitem 22-i? *

Copy and paste relevant sections from the manuscript (include quotes in quotation marks "like this" to indicate direct quotes from your manuscript), or elaborate on this item by providing additional information not in the ms, or briefly explain why the item is not applicable/relevant for your study

"The investigation of patient satisfaction after a hospital stay resulted in several differences when comparing two modes of questionnaire administration, self-reported internet completion or telephone interview. The comparison between these modes of administration may be discussed according to three topics: response rate, questionnaire reliability, and satisfaction scores.

The response rate observed in the group of patients randomized in the Internet group (39%) was much lower than that observed in the group of patients randomized in the Telephone group (88%). Such a difference might have resulted to unbalancing initial comparability of responders in the two groups even if Table 1 indicates that baseline variables are similar in the responders of the two groups.

Unsurprisingly, the observed difference between the two groups in terms of participation rate is in accordance with previous results issued from the same cohort focussing on patient satisfaction in regards to the hospital discharge process [23]. The difference between the participation rates observed with the two administration modes of the survey might be due, at least in part, to the fact that it is easier to ignore an e-mail than a phone call scheduled at a date chosen by the patient. The participation rates observed in our study are also similar to those reported by Harewood et al who investigated patient satisfaction with endoscopy and observed a response rate of 34% and 78% in the Internet and Telephone group, respectively [18].

Excepted those for the hospital room convenience dimension which raise concerns, the values of Cronbach's alpha were satisfactory for all dimensions investigated and for the global satisfaction score, favouring the conduction of surveys with this questionnaire using either administration mode. Besides, interestingly, considering all 6 dimensions of the questionnaire, the values of Cronbach's alphas were always higher in the Internet group than in the Telephone group, with a statistical significance observed for two dimensions (global care, hospital room convenience) and for the global satisfaction score. Here, the adjunction of an interviewer in the Telephone group, (as compared to self-completion in the Internet group) might be considered as an undesired burden disturbing initial signal.

The observed score differences between the Internet and Telephone group (see Table 3) are contrasted, depending on the dimension investigated. On the one hand, considering hospital room convenience and hospital catering dimensions, telephone and internet modes of administration resulted in very similar satisfaction scores, and the difference was only slight when considering global [illegible]

22-ii) Highlight unanswered new questions, suggest future research

Highlight unanswered new questions, suggest future research.

	1	2	3	4	5	
subitem not at all important	○	○	○	○	○	essential

Does your paper address subitem 22-ii?

Copy and paste relevant sections from the manuscript (include quotes in quotation marks "like this" to indicate direct quotes from your manuscript), or elaborate on this item by providing additional information not in the ms, or briefly explain why the item is not applicable/relevant for your study

"... although it is worth mentioning that corresponding effect sizes never exceeded 0.35, a value below the medium threshold proposed by Cohen [29]. Such an observation raises a general comment on the surveys made in this domain. Those surveys are deployed for investigating patient satisfaction on hospital services, for bringing into light the elements which require improvements, for assessing evolution with time, and French authorities require the analysis of 120 patients per medical centre each year. In such a context, our finding, in the present study, that a mean difference of 7 points based on a sample size of 498 responders is modest in terms of effect size, suggests that potential improvements on patient satisfaction are very difficult to evidence: dynamic trends within a given centre from one year to another should be interpreted with great caution and must take into account the underlying variability of the scores, and a similar caution should be required in the interpretation of differences between centres. A potential explanation for the higher scores observed in the Telephone group is that a patient might be more reluctant to provide low scores to an interviewer (moreover potentially identified as a member of the hospital staff) than when completing a strict anonymous form via the Internet. In addition, the distribution of the delay between hospital discharge and questionnaire completion was more variable in the internet group. Therefore, one cannot exclude that the corresponding difference in delay distributions between the two [illegible]

20) Trial limitations, addressing sources of potential bias, imprecision, and, if relevant, multiplicity of analyses

20-i) Typical limitations in ehealth trials

Typical limitations in ehealth trials: Participants in ehealth trials are rarely blinded. Ehealth trials often look at a multiplicity of outcomes, increasing risk for a Type I error. Discuss biases due to non-use of the intervention/usability issues, biases through informed consent procedures, unexpected events.

	1	2	3	4	5	
subitem not at all important	○	○	○	○	○	essential

Does your paper address subitem 20-i? *

Copy and paste relevant sections from the manuscript (include quotes in quotation marks "like this" to indicate direct quotes from your manuscript), or elaborate on this item by providing additional information not in the ms, or briefly explain why the item is not applicable/relevant for your study

"The response rate observed in the group of patients randomized in the Internet group (39%) was much lower than that observed in the group of patients randomized in the Telephone group (88%). Such a difference might have resulted to unbalancing initial comparability of responders in the two groups even if Table 1 indicates that baseline variables are similar in the responders of the two groups."

21) Generalisability (external validity, applicability) of the trial findings

NPT: External validity of the trial findings according to the intervention, comparators, patients, and care providers or centers involved in the trial

21-i) Generalizability to other populations

Generalizability to other populations: In particular, discuss generalizability to a general Internet population, outside of a RCT setting, and general patient population, including applicability of the study results for other organizations

	1	2	3	4	5	
subitem not at all important	○	○	○	○	○	essential

Does your paper address subitem 21-i?

Copy and paste relevant sections from the manuscript (include quotes in quotation marks "like this" to indicate direct quotes from your manuscript), or elaborate on this item by providing additional information not in the ms, or briefly explain why the item is not applicable/relevant for your study

"the fact that this questionnaire is yet restricted to France constitutes a limitation of the study."
"In conclusion, our study shows that the lower response rate observed with the internet mode of administration than that observed with the Telephone mode of administration must be balanced with other positive features associated with the Internet. Using the latter mode of administration that has a potential lower cost than Telephone [14], the quality of satisfaction estimates is likely improved since the potential veil of a telephone interviewer is discarded, patients express more freely and are more likely to rate their satisfaction about hospital stay with lower scores."

21-ii) Discuss if there were elements in the RCT that would be different in a routine application setting

Discuss if there were elements in the RCT that would be different in a routine application setting (e.g., prompts/reminders, more human involvement, training sessions or other co-interventions) and what impact the omission of these elements could have on use, adoption, or outcomes if the intervention is applied outside of a RCT setting.

1 2 3 4 5

subitem not at all important ○ ○ ○ ○ ○ essential

Does your paper address subitem 21-ii?

Copy and paste relevant sections from the manuscript (include quotes in quotation marks "like this" to indicate direct quotes from your manuscript), or elaborate on this item by providing additional information not in the ms, or briefly explain why the item is not applicable/relevant for your study

"Using the latter mode of administration that has a potential lower cost than Telephone [14], the quality of satisfaction estimates is likely improved since the potential veil of a telephone interviewer is discarded, patients express more freely and are more likely to rate their satisfaction about hospital stay with lower scores."

OTHER INFORMATION

23) Registration number and name of trial registry

Does your paper address CONSORT subitem 23? *

Copy and paste relevant sections from the manuscript (include quotes in quotation marks "like this" to indicate direct quotes from your manuscript), or elaborate on this item by providing additional information not in the ms, or briefly explain why the item is not applicable/relevant for your study

"Trial registration number. Clinicaltrials.gov NCT01769261; http://clinicaltrials.gov/ct2/show/NCT01769261. Archived at http://www.webcitation.org/6ZDF5lA41"

24) Where the full trial protocol can be accessed, if available

Does your paper address CONSORT subitem 24? *

Cite a Multimedia Appendix, other reference, or copy and paste relevant sections from the manuscript (include quotes in quotation marks "like this" to indicate direct quotes from your manuscript), or elaborate on this item by providing additional information not in the ms, or briefly explain why the item is not applicable/relevant for your study

NCT01769261 Clinicaltrials.gov
https://clinicaltrials.gov/ct2/show/record/NCT01769261

25) Sources of funding and other support (such as supply of drugs), role of funders

Does your paper address CONSORT subitem 25? *

Copy and paste relevant sections from the manuscript (include quotes in quotation marks "like this" to indicate direct quotes from your manuscript), or elaborate on this item by providing additional information not in the ms, or briefly explain why the item is not applicable/relevant for your study

"The Assistance Publique–Hôpitaux de Paris (Département de la Recherche Clinique et du Développement) was the trial sponsor.
The SENTIPAT study was funded by grant AOM09213 K081204 from Programme Hospitalier de Recherche Clinique 2009 (Ministère de la Santé).
The sponsor and the funders had no role in study design, data collection and analysis, decision to publish, or preparation of the

X27) Conflicts of Interest (not a CONSORT item)

X27-i) State the relation of the study team towards the system being evaluated

In addition to the usual declaration of interests (financial or otherwise), also state the relation of the study team towards the system being evaluated, i.e., state if the authors/evaluators are distinct from or identical with the developers/sponsors of the intervention.

	1	2	3	4	5	
subitem not at all important	○	○	○	○	○	essential

Does your paper address subitem X27-i?

Copy and paste relevant sections from the manuscript (include quotes in quotation marks "like this" to indicate direct quotes from your manuscript), or elaborate on this item by providing additional information not in the ms, or briefly explain why the item is not applicable/relevant for your study

"none declared"

About the CONSORT EHEALTH checklist

As a result of using this checklist, did you make changes in your manuscript? *

- ○ yes, major changes
- ● yes, minor changes
- ○ no

What were the most important changes you made as a result of using this checklist?

Methiod section, description of the trial on which this ancillary study is based

How much time did you spend on going through the checklist INCLUDING making changes in your manuscript *

8 hours

As a result of using this checklist, do you think your manuscript has improved? *

- ◯ yes
- ◯ no
- ◉ Autre : only very slightly

Would you like to become involved in the CONSORT EHEALTH group?

This would involve for example becoming involved in participating in a workshop and writing an "Explanation and Elaboration" document

- ◯ yes
- ◉ no
- ◯ Autre :

Any other comments or questions on CONSORT EHEALTH

Equator guidelines are undoubtedly helpful when writing scientific articles (I am familiar with CONSORT, STROBE, CHEERS and PRISMA guidelines). I understand your concerns about specific patterns relating to trials in e-health. However, I feel that much more time was required for completing the present form than the time that would have been required for completing consort extension for non pharmacological treatments (the "nearest" equator guideline that would correspond to present study design). A substantial

STOP - Save this form as PDF before you click submit

To generate a record that you filled in this form, we recommend to generate a PDF of this page (on a Mac, simply select "print" and then select "print as PDF") before you submit it.

When you submit your (revised) paper to JMIR, please upload the PDF as supplementary file.

Don't worry if some text in the textboxes is cut off, as we still have the complete information in our database. Thank you!

Final step: Click submit !

Click submit so we have your answers in our database!

Envoyer

N'envoyez jamais de mots de passe via Google Forms.

Fourni par

Ce contenu n'est ni rédigé, ni cautionné par Google.

Signaler un cas d'utilisation abusive - Conditions d'utilisation - Clauses additionnelles
